# Behavioural factors matter for the adoption of climate-smart agriculture

**DOI:** 10.1038/s41598-023-50264-4

**Published:** 2024-01-08

**Authors:** Martin Paul Jr Tabe-Ojong, Marvin Ebot Kedinga, Bisrat Haile Gebrekidan

**Affiliations:** 1 World Bank, Washington, DC USA; 2 Disaster Management Training and Education Centre (DiMTEC) for Africa, University of the Free State, UFS Internal 66, P.O. Box 339, Bloemfontein, South Africa; 3 University of Applied Sciences, Sankt Augustin, Germany; 4 Intstitute for Food and Resource Economics, University of Bonn, Bonn, Germany

**Keywords:** Psychology, Climate sciences, Environmental sciences, Environmental social sciences

## Abstract

Increasing agricultural productivity while ensuring environmental sustainability are two important targets in achieving the sustainable development goals under climatic shocks. In this regard, different climate-smart agricultural (CSA) practices have been recommended and promoted to meet these goals and targets. However, the adoption of these practices remains low and variable. For the most part, low adoption has been attributed to external factors. Behavioural and psychological factors also matter but have received little empirical and policy attention. In this study, we examine the relationship between aspirations, aspiration gaps, and the adoption of CSA practices such as crop rotation, intercropping, fallowing, and organic soil amendments. Employing parametric and non-parametric estimation techniques on a pooled farm household survey from Cameroon and Kenya, we show that aspirations are associated with the use of crop rotation and organic soil amendments. We also investigate the theorized non-monotonic inverse U-shaped relationship between aspiration gaps and investments. We find evidence of this relationship for the adoption of these CSA practices, suggesting an aspiration failure for smallholder farmers. These results imply that aspirations that are ahead but not too far ahead of the current state serve as the best incentives for stimulating the adoption of CSA practices. Employing the multivariate probit model, we further highlight interdependencies in the use of these CSA practices. Specifically, we underscore significant complementarities, suggesting the bundled use of these practices. Overall, the analysis demonstrates that aspirations matter for farmer decision-making with many implications for agricultural, food, and environmental policies.

## Introduction

Increasing agricultural productivity while ensuring environmental sustainability are two important targets in achieving the sustainable development goals, especially under climatic shocks^1^. In this regard, the promotion of climate-smart agriculture (CSA) has been a priority on development and policy agenda^2–5^. CSA has been highlighted to offer the triple wins of^1^ increasing productivity with ensuing income and welfare implications;^2^ reducing the emission of greenhouse gases; and^3^ building resilience to climate shocks. CSA practices are context-specific, that is, different practices are used under different farming systems and agro-ecological conditions with differential implications^6^. Despite their proven benefits in meeting these productivity and sustainability goals, their adoption has been less than optimal and highly variable^7^. Several external factors such as liquidity and credit constraints, missing markets, and lack of information have been highlighted to be some constraints farmers are facing in adopting these CSA practices^8–12^.

The role of behavioural and psychological factors has been overlooked, although they are increasingly being recognised as important in economic decision making^13,14^. These behavioural factors have been described as more internal constraints to decision making^15^. They have the potential to push individuals to believe that they do not have the capabilities and control for being successful in many aspects of life^16^ including investments in agricultural production^17,18^. One behavioural factor that is receiving growing interest is the concept of aspirations which has been shown to positively influence future-oriented economic behaviours^19–21^. However, aspirations may backfire if they are too high and do not match the available investment capacities^22,23^. This relationship constitutes the theorized non monotonic inverse U-shaped relationship between the aspirations gap (the difference between current and aspired levels) and investment^22,23^. This implies that aspirations should be large enough to incentivize but not overly large to induce frustration and resignation arising from unattainable perceived efforts.

In this study, we examine the relationship between aspirations and the adoption of CSA practices such as crop rotation, intercropping, fallowing, and organic soil amendments. These practices are agronomic practices that build resilience to climate change and support environmental sustainability while increasing agricultural production. Intercropping which involves the cultivation of legume crops and cereals, has been described as pro-poor and environmentally friendly as it provides both nutritional and environmental benefits for poor households^6^. It also represents as aspect of crop diversification which is important for dietary diversity and food consumption^6^. This is also the case for organic soil amendments which is associated with food security^24^. Some of the crops cultivated under intercropping have also been highlighted to contribute to carbon sequestration in many drylands of Africa^25^. Crop rotation has been highlighted to reduce the build-up of pests and diseases which are increasingly common under climatic shocks^26^. Fallowing also offers similar advantages when it comes to the management of pests and weeds, besides enabling nutrient restoration^27^. We also evaluate the theorized non-monotonic relationship between aspirations and investments by further examining the relationship between the aspiration gap and investment in CSA practices at the extensive margin. While aspirations are goals/targets individuals set to achieve in the future, an aspiration gap refers to the difference between the current and aspired levels of individuals. It is this gap and not aspirations per se that incentivises investments^20,22,23^.

Given the plausible importance of adopting these practices as a bundle, we examine interdependencies by assessing potential complementarities and/or trade-offs in the use of these practices. Our analysis relies on a pooled farm household survey from smallholder farmers in Cameroon and Kenya. We employ different empirical strategies including parametric (linear probability model, multivariate probit model, Ordered Probit model and Poisson regression model) and nonparametric regressions (loess smoothing). Given the interest in finely testing the supposed inverse U-shaped relationship, we also perform some specialised U-shaped tests following the procedures of^28^. We obtain three key results: First, aspirations are positively associated with the use of crop rotation and organic soil amendments under the linear set-up as well as under the multivariate probit set-up where we assume interdependence of the various CSA practices. These estimates are further robust to both the Ordered Probit model and the Poisson regression model. Second, we report the existence of an inverse U-shaped relationship between the aspiration gap and the adoption of CSA practices. This suggests the existence of aspiration failure in the adoption of CSA practices. Aspiration failure here refers to both the failed capacity to aspire (low aspirations) and frustration from high aspirations^19^. In the last place, we establish complementarities in the use of these CSA practices, which corroborates the precedence of bundling these practices for best outcomes^6^.

Our paper offers several contributions to different strands of the literature on aspirations and future-oriented economic behaviours as well as the adoption of sustainable agricultural practices. To begin with, we add empirical insights to studies that have established a positive association between different behavioural factors and the adoption of sustainable farm practices^13,29,30^. Our key addition here is looking at aspirations which has so far not been linked to CSA. The second addition relates to confirming the non-monotonic relationship between aspirations and investments in the case of the adoption of CSA practices at the extensive margin. Although these practices can be described as low input short term investments, they offer significant productivity and sustainability gains both in the short and long terms^2,6^. Previous studies on this relationship exists but they are mostly focused on other socio-economic outcomes such as educational achievements, savings, financial decisions, and real estate investments^31–35^. Investments in agricultural technologies is understudied in terms of their relationship with aspirations (gap). One exception is work from Ecuador that links the aspiration gap to farm investments such renovations and the use of fertilisers^18^. We build on this analysis and add more insights on the inverted U-shaped relationship for the case of CSA practices.

The third contribution comes from our focus on CSA practices. As earlier highlighted, these practices have immense implications for a range of productivity and environmental goals especially under climatic shocks. Insights from this analysis has implications for achieving some of the United Nations Sustainable Development Goals (SDGs) for the 2030 Sustainable Development Agenda. Particularly, these insights relate to poverty (SDG 1), hunger (SDG 2), decent work and economic growth (SDG 8), reducing inequalities (SDG 9), responsible consumption and production (SDG 12), climate action (SDG 13), life below water (SDG 14) and life on land (SDG 15). Here, we also provide and strengthen the insight that CSA practices should be adopted as a bundle in line with the significant complementary relationships that we underscore. The final contribution stems from the external validity of our findings. We provide evidence beyond a single case study and additionally provide cross-country insights from two different production zones and farming systems in Cameroon and Kenya.

The rest of the article is structured as follows. Section two provides a brief conceptual link between aspirations, aspiration gaps and investments. The empirical strategy is highlighted in section three while the farm household survey, variable description and measurements are presented in section four. In section five, both descriptive and empirical results are presented and discussed, and the article ends with some remarks and policy implications in section six.

## Aspirations, aspiration gap and investments

Aspirations are goals to which individuals invest time, money, and effort for their attainment^36^. They are also personally meaningful objectives that are supported by evidence of positive results that are intended to motivate individuals^37^. Since the pioneering work of the anthropologist Arjun Appadurai, economists have taken keen interests in modelling aspirations. In his seminal paper,^19^ came forward with the concept of the ‘capacity to aspire’ which he related to the future-oriented logic of development. Aspirations internalises constraints but also factors in efforts that may be put to overcome these constraints in a bid to reach the aspired levels^15^. These efforts may take the form of investments and savings that have the potential to increase potential future payoffs.

Further conceptualizing this,^20^ came forward with additional concepts such as the aspiration gap and aspiration failure. The aspiration gap is defined as the difference/distance between the current and the aspired level of an individual. According to^20^, it is this distance (gap) and not aspirations per se that influences future-oriented behaviours. When the gap is low (which may signify low aspirations), individuals may internalize their resource constraints and not see a possibility in any investment especially given the little amount of effort required. This is referred to as aspiration fatalism which constitutes part of aspiration failure as low aspirations do not incentivise investments^17,38^. Under this scenario, individuals can be described as having a low capacity to aspire which reduces their navigational capacity^16^. At any point after this zone which^22^ describe as the ‘satisfaction zone’, an increase in aspirations may result to investments. Under this zone, investments would be made up to a reference aspiration threshold point beyond which little or no investments would be made. This is the scenario when the gap is large, making individuals to doubt their ability in reaching their aspired level given the envisaged high effort involved. (It is important to recognize that an aspirations gap could also take the form of not seeing agriculture (CSA) as the most viable way of reaching the aspiration level (in this case, income). Individuals may be pursuing their aspirations through non-farm activities perhaps) In this case, they may quickly give up to resignation and become frustrated by their limited ability to meet their aspirations. This is also referred to as aspiration frustration which is also a form of aspiration failure. Thus, both aspiration fatalism and aspiration frustration constitute aspiration failure^22,23^.

Both low and very high aspiration gaps may not lead to investments. This behaviour constitutes the theorized non-monotonic relationship between the aspiration gap and investments^22,23^. Thus, it goes without saying that aspirations should be large enough to incentivize but not very large to induce frustration. In this regard, the best aspirations would be aspirations that are slightly above the current levels, such that individuals can navigate local realities and utilise the available resources around them to meet their aspirations. When aspirations are too high, they may be constrained by material lack despite putting the necessary efforts to attain their aspirations. Thus, when aspirations are (too) low, providing big push interventions such as cash transfers may be required to get individuals to an optimal level to build their aspirations and get them on a comfortable base. Previous studies have identified exposure to role models, nudging, training, membership in various social groups, and social relief support to be important ways of increasing aspirations^39–43^. Beyond these social factors, circumstantial factors such as income also matter and could either reduce or induce higher aspirations^21,40,44^.

## Methodology

### Empirical estimation

We are interested in understanding the relationship between aspirations and the adoption of CSA practices such as crop rotation, intercropping, fallowing, and the use of organic soil amendments. We are also interested in examining the theorized inverted U-shaped relationship between the aspiration gap and the adoption of CSA practices. For the first research objective, we estimate regression equations of the form:

1 $${Y}_{iv}= {\beta }_{0}+ {\beta }_{1}{A}_{iv}+ { {{\varvec{\beta}}}_{2}{{\varvec{X}}}_{{\varvec{i}}{\varvec{v}}}+ l}_{v}+{ \varepsilon }_{iv}$$

where ${Y}_{iv}$ represents the outcome variable (crop rotation, intercropping, fallowing, organic soil amendments) of farmer $i$ in village $v$. Our variable of interest is ${A}_{iv}$ which represents the income aspirations of households. Its parameter estimate is ${\beta }_{1}$. ${{\varvec{X}}}_{{\varvec{i}}{\varvec{v}}}$ represents a vector of control variables such as age and educational level, household size, household head is male, credit access, extension access, cooperative membership, off-farm employment, and wealth status of farmers.${l}_{v}$ is the village fixed effect which controls for village level heterogeneities. ${\varepsilon }_{iv}$ is the random error term. Standard errors are clustered at the village level.

For the second objective where we are interested in examining the inverse U-shaped relationship between the aspiration gap and CSA practices, we perform both parametric and non-parametric regressions. The difference in both regressions is based on functional form assumptions as we show below. For the parametric estimation, we estimate the following regression equation:

2 $${Y}_{iv}= {\theta }_{0}+ {\theta }_{1}{G}_{iv}+ {{\theta }_{2}{{G}_{iv}}^{2}+ {{\varvec{\theta}}}_{3}{{\varvec{Z}}}_{{\varvec{i}}{\varvec{v}}}+ v}_{v}+{ u}_{iv}$$

Here, our variables of interest are ${G}_{iv}$ and ${{G}_{iv}}^{2}$ which represent the aspiration gap and the square of the aspiration gap. A positive value of ${\theta }_{1}$ and a negative value of ${\theta }_{2}$ may suggest the presence of an inverse U-shaped relationship. However, this may be misleading^28^. This is common when the true relationship is convex but monotone over different data values. To confirm this, we perform some U-shaped tests where we verify whether the relationship is increasing and decreasing at high and low values within the interval^28^. These tests serve as a first robustness check on the estimated U-shaped relationship. We estimate various slopes at both maximum and minimum turnings points, Sasabuschi p-values and the Fieller 95% confidence intervals. Despite all these, we still perform a non-parametric regression to further confirm these earlier regressions.

For the non-parametric regression, we estimate the following regression specification:

3 $${Y}_{iv}= {\sigma }_{0}+ f({G}_{iv}) {+ {{\varvec{\sigma}}}_{3}{{\varvec{Z}}}_{{\varvec{i}}{\varvec{v}}}+ e}_{v}+{ v}_{iv}$$

The same notations apply, with ${e}_{v}$ representing the village fixed effects, ${v}_{iv}$ is the random error term and ${{\varvec{Z}}}_{{\varvec{i}}{\varvec{v}}}$ is a vector of control variables. One difference between Eqs. (2) and (3) is how the square of the aspiration gap enters the third equation. The non-parametric estimation (loess smoothing) is more flexible and does not impose a prior functional form on how this enters the model. This estimation could additionally serve as the second robustness check on the parametric estimation and the U-shaped tests. Given that all our outcome variables are dichotomous, we employ the linear probability model instead of the regular probit/logit model. Since we are implicitly interested in the relationship between aspirations, aspirations gap and investments, a linear specification is preferable as it avoids identification by functional form. Moreover, it is easier and less cumbersome to interpret. That notwithstanding, we also estimate some non-linear models and show that the findings are in the same direction.

Specifically, we estimate a multivariate probit (MVP) model instead of the standard probit and/or logit models. The choice in using the MVP model is based on the premise that the adoption of these CSA practices may not be independent. That is, the adoption of crop rotation may be related to intercropping as well as to fallowing and the use of organic soil amendments. The MVP model accommodates these interdependencies^45^. It specifies whether these practices are substitutes or complements by allowing for flexible correlations in the error terms associated the use of these CSA practices. Additionally, it also shows the relationship between aspirations, aspirations gap and the adoption of these CSA practices. The estimated MVP model consist of four binary type equations representing the four CSA practices: crop rotation, intercropping, fallowing, and the use of organic soil amendments The latent and ensuing equations resulting from this can be defined as:

4 $${Y}_{ivn}^{*}= {{A}_{ivn}{\beta }_{1}+\boldsymbol{ }{\varvec{X}}}_{ivn}{\prime}{{\varvec{\beta}}}_{2}+ {\varepsilon }_{ivn} \;\mathrm{ n }= 1, 2, 3, 4$$

5 $${Y}_{ivn}= \left(\genfrac{}{}{0pt}{}{1 if\; {Y}_{ivn}^{*}>0}{0 otherwise}\right)$$

where ${Y}_{ivn}^{*}$ is the latent variable representing utility differences in the adoption of these CSA practices. Households would adopt the CSA practices if their expected net benefit of adoption is greater than zero. The adoption of these CSA practices as we have highlighted earlier is a linear combination of both observable (${{\varvec{X}}}_{ivn}{\prime})$ and unobservable factors (${\varepsilon }_{ivn}$). All variables are as previously defined. Our interest lies in both the role of observable characteristics that drive the use of CSA as well as unobservable factors that account for interdependencies in the error terms of adoption of these CSA practices. The MVP model computes an error correlation matrix which is summarises the various correlations as either positive or negative. While a positive correlation signifies complementarities, a negative correlation represents substitutability^46^.

### Robustness checks

Given that farmers adopt these CSA practices as a bundle, we estimate some additional regressions to reflect this reality. First, we generate a count of all the CSA practices adopted by farmers and employ a Poisson regression model to estimate the relationship between aspirations and the number of CSA practices adopted by farmers. In the same vein, given we know the number of practices adopted by each farmer, we further use an ordered probit model to also estimate the relationship between aspirations and the CSA combinations.

### Farm household survey data

This analysis is based on two cross-sectional surveys conducted in the Littoral region of Cameroon and the Baringo county of Kenya. The surveys used identical questionnaires and were conducted with similar objectives of understanding behavioural constraints facing farmers in rural areas. Both surveys followed a multistage sampling technique. In the first place, five representative districts were purposively chosen (3 in Kenya and 2 in Cameroon). From these districts, 74 (35 in Kenya and 39 in Cameroon) villages were randomly selected using the probability proportional to size sampling approach. These villages then formed the primary sampling unit from which households were randomly selected. In these villages, 13–17 households were randomly selected and interviewed. 530 households were interviewed in Kenya while 582 households were interviewed in Cameroon. Interviews were carried out in the native languages of the study areas. The interviews were carried out by a group of enumerators supervised by one of the authors. Enumerators were well trained prior to the commencement of the data collection process to enable them to have a firm grip of the questionnaire. This is important given that aspirations can easily be confused with other nuanced behavioural concepts such as expectations and non-cognitive skills. In additional to the household surveys, informal discussions were conducted with village chiefs and community leaders to obtain anecdotal insights on the context and production environments of the survey sites.

The survey collected information on various farm and household characteristics such as age and educational level of the household head, income level, household size, off-farm employment, wealth status, access to institutional services such as agricultural extension, credits, and membership in agricultural cooperatives. Households in both countries are mixed crop farmers producing both crops and rearing livestock. The main annual crops produced in the study areas of both countries are maize, beans, groundnuts as well as fruits and vegetables. In Cameroon, households are also cultivating oil palm which is both used for food and cash purposes.

### Measurement of outcome variables

We have four outcome variables: crop rotation, intercropping, fallowing, and organic soil amendments which are all various aspects of climate smart agriculture^2–5^. Crop rotation is the practice of sequentially planting different crops on the same plot for various soil biological activities and pest/weed management. Crop rotation as a CSA practice offers several benefits such as improving soil health and fertility, as well as reducing the build-up of pests and diseases. With regards to the latter, recent analysis shows that crop rotation which constitutes some aspects of diversification reduces the incidence of pests and their impacts^26^. Intercropping is the practice of growing two or more different crops together. In this context, it is mainly about legume and cereal intercropping which has the probability to increase soil fertility through biological nitrogen fixation from the legume^6^. Fallowing refers to keeping a farmland untilled or unsown for some time to allow soil fertility regeneration and other biological functions. Finally, organic soil amendments refer to the use of green and animal manure to improve the fertility of the soil and it has been argued to be important for food security^24^. All these variables are captured as dichotomous variables. Farmers were asked whether they used these practices in the most recent agricultural season. Answers to these questions were recorded as either yes for farmers using these practices, taking the value of 1 and zero otherwise.

### Measurement of variables of interest

We have three variables of interest: aspirations, aspiration gap and the square of aspiration gap. To measure all these variables, we relied on the well tested and validated way of measuring aspirations in rural settings^36^. It has also been used in other aspiration studies^31,33,44^. It is a direct way of asking individuals about their aspirations. It begins by asking individuals their current income status and then their income aspirations. Our first variable of interest is directly gotten from the latter question. The former question is important in obtaining the aspiration gap which is the second variable of interest. The aspiration gap is obtained by taking the difference between the income aspiration and the current income. This difference is then divided by the income aspirations. This way of computation bounds the values of the aspiration gap between 0 and 1 and enables comparison across households given differences in incomes. From these variables, we generate the aspiration gap squared by squaring the aspirations gap. Given that the income and aspiration measure are all recorded differently using the local currencies of the countries, we converted all these variables to the US dollar ($) Purchasing Power Parity (PPP) using exchange rates extrapolated from the 2017 International Comparison Program (ICP) of the World Bank. This is important to enable meaningful cross-country comparisons. In the regression models, we transform these variables using the inverse hyperbolic sine transformation.

### Ethical considerations

The authors confirm that the household survey was carried out in accordance with relevant guidelines and regulations. The study was also approved by the Center for Development Research Ethics Committee at the University of Bonn and was found to be ethically sound. Additionally, informed consent was obtained from all interviewed households prior to the start of the survey.

## Results and Discussion

### Descriptive insights

We present the summary statistics of some of the regression variables especially the outcome variables (Table S1 in the supplementary material). We differentiate the use of these practices by country to have an early understanding of the differences in the two countries.

**Figure 1:**
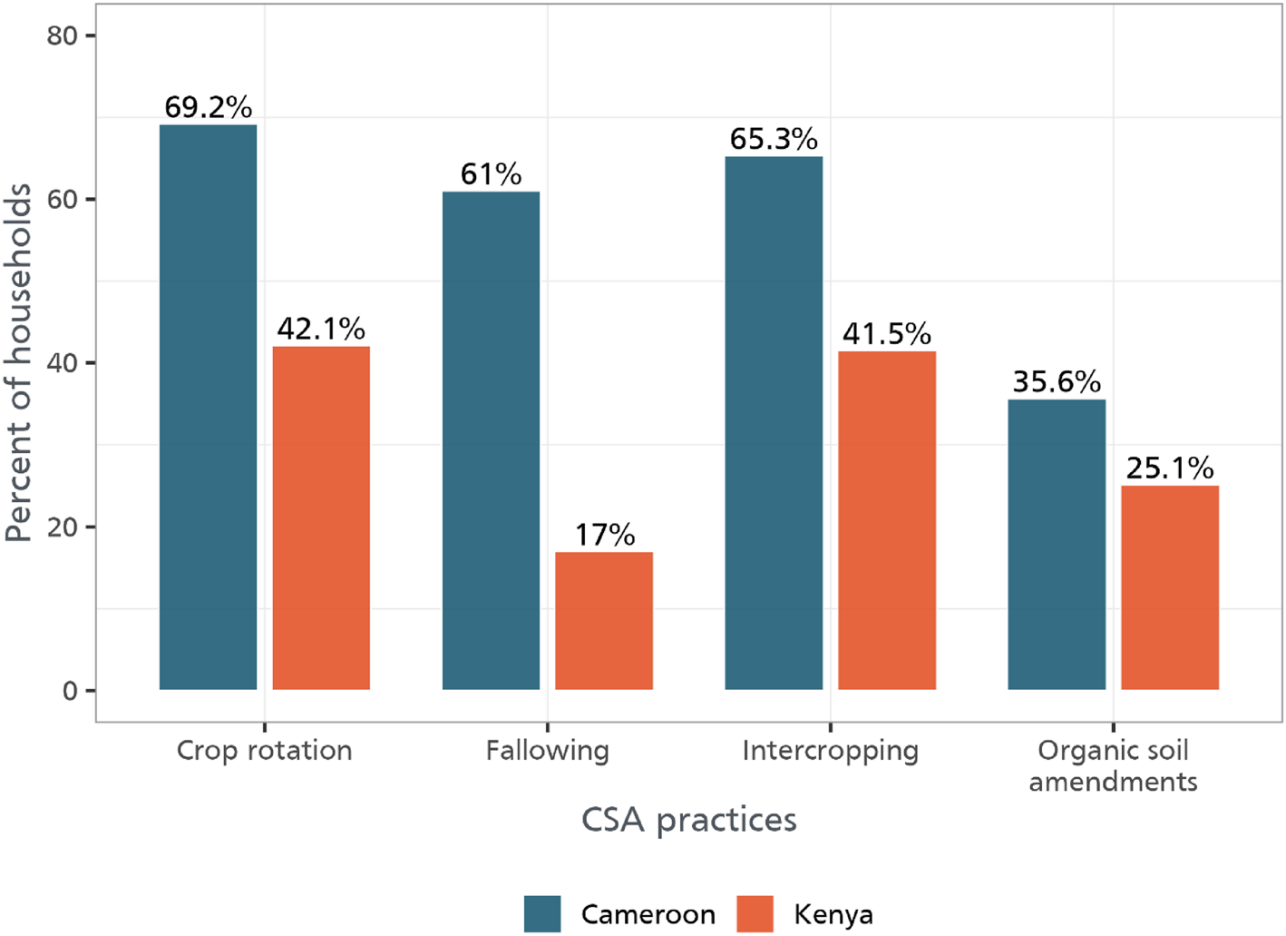
CSA practices by country.

**Figure 2:**
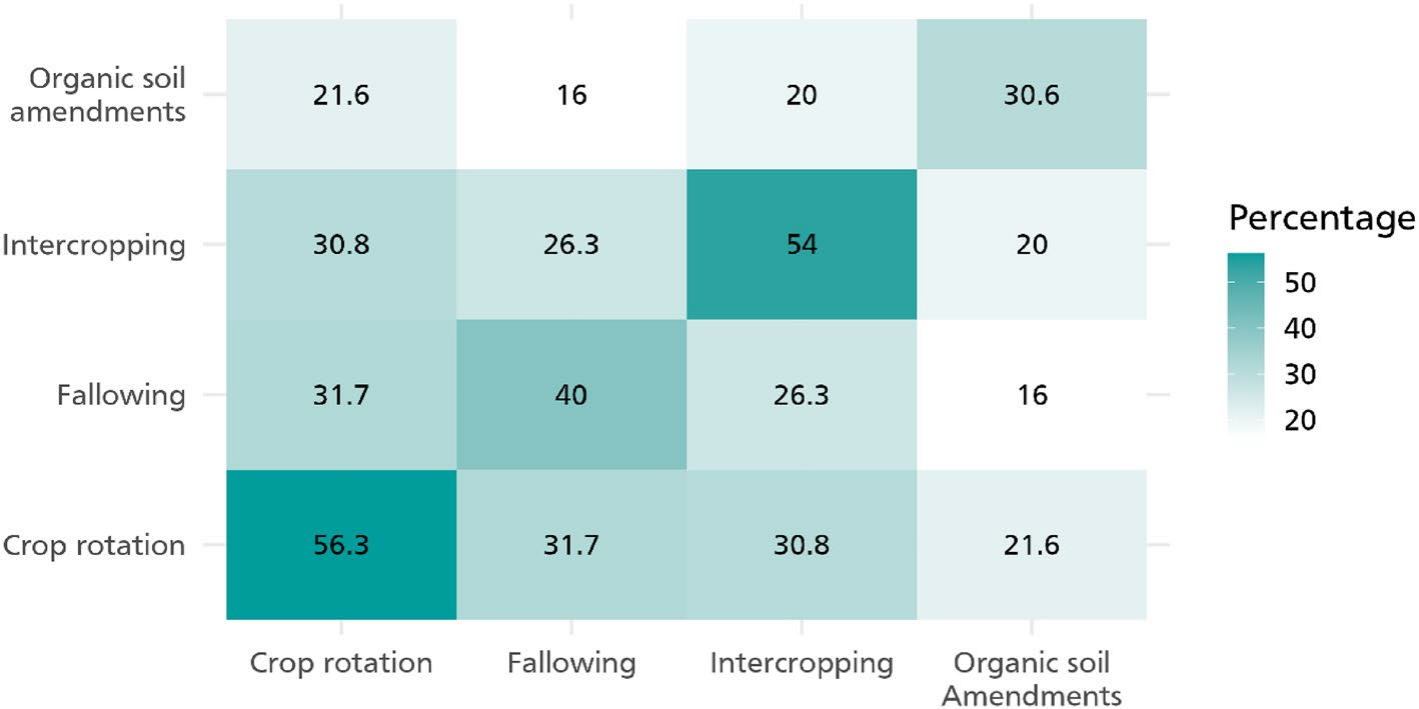
Pairwise adoption of CSA practices. The plot represents the pairwise adoption percentage of CSA practices across households. Each tile represents a pair of practices. For example, the tile at the intersection of "Crop rotation" and "Intercropping" represents the percentage of households that adopted both "Crop rotation" and "Intercropping". The color of each tile indicates the percentage of adoption. Darker tiles signify a higher percentage of adoption, as denoted by the color gradient legend. The numbers in the tiles are the actual percentages of households adopting both practices in the corresponding pair.

As shown in Fig. 1, farmers in both Cameroon and Kenya are adopting crop rotation, intercropping, fallowing, and organic soil investments. However, adoption rates are higher in Cameroon than in Kenya. About 42% of sampled households in Kenya are practicing crop rotation as opposed to about 63% in Cameroon. This is also the case of intercropping which follows a similar trend. Fallowing is only used by about 17% of farmers in the study region in Kenya in comparison to closely 61% in Cameroon. Figure 2 also shows the pairwise adoption of these practices. Given that we also run some count and ordered probit models, Table S1 also shows the percentage of farmers adopting 1, 2, 3 or all the practices. Farmers are largely into agricultural production where they cultivate both food and cash crops which earn them income. Income is measured and reported monthly.

We also show some of the other household and farm characteristics where we again differentiate by country (Table S1). Households report income aspirations of about $1392 in purchasing power parity terms. They also have incomes of about $252, still in purchasing power parity terms. The difference of these two variables gives us the aspiration gap. Farmers report a gap of 0.73 and aspiration gap square of 0.58 which are both similar in Cameroon and Kenya. Beyond their agricultural activities, households participate in off-farm employment. They are also members of social groups such as agricultural cooperatives and have access to extension as well as financial services such as credits. About 25% of the household heads are females and households have about 5 members on average.

### Empirical insights

Here, we present the results of both the parametric and non-parametric estimations. We also present the u-test results (full results in Table S4 in the supplementary material). Additionally, we show the estimates of the multivariate probit model where we establish potential interdependencies. We also present the results of the Poisson regression and Ordered Probit model.

#### Parametric estimation results

##### Aspirations and adoption of CSA practices

**Figure 3:**
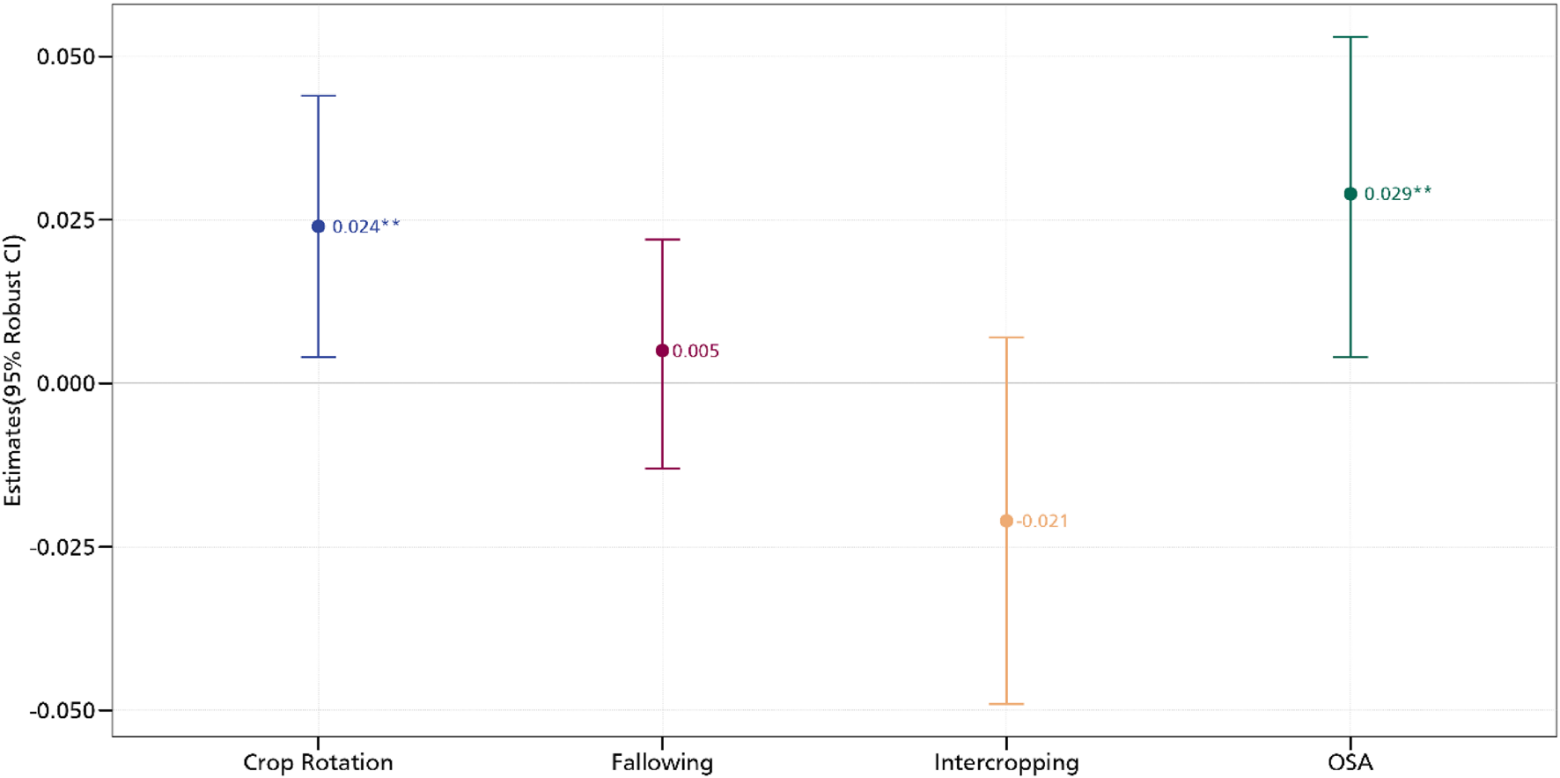
Estimates of aspirations and CSA practices. OSA is organic soil amendments. The models are estimated with additional controls such as age and educational level, whether household head is male, cooperative membership, extension access, credit access, household size, off-farm employment, and wealth index. *** *p* < 0.01, ** *p* < 0.05, * *p* < 0.1. Full results are presented in Table S2 in the supplementary material.

We present the results of the association between aspirations and the CSA practices in Fig. 3. In all the models, we control for village fixed effects and cluster the standard errors at the village level. Aspirations exhibit a positive association with crop rotation. Aspirations are associated with a 2.4 percentage point increase in the adoption of crop rotation. A similar insight is also obtained for the use of organic soil amendments where we observe a 2.9 percentage point increase. We do not find any relationship between aspirations and intercropping as well as fallowing. The insights here speak to the positive correlation between aspirations and CSA practices. These findings are in order and support previous findings that have established the role of behavioural factors in driving sustainable and climate-resilient farm practices^13,29,30^. Particularly, the positive association between aspirations and crop rotation support an earlier analysis that found a similar relationship between aspirations and the use of preventive measures against pests^29^. Crop rotation as a CSA practice offers several benefits such as improving soil health and fertility, as well as reducing the build-up of pests and diseases. With regards to the latter, recent analysis shows that crop rotation which constitutes some aspects of diversification reduces the incidence of pests and their impacts^26^.

##### Aspiration failure and adoption of CSA practices

**Figure 4:**
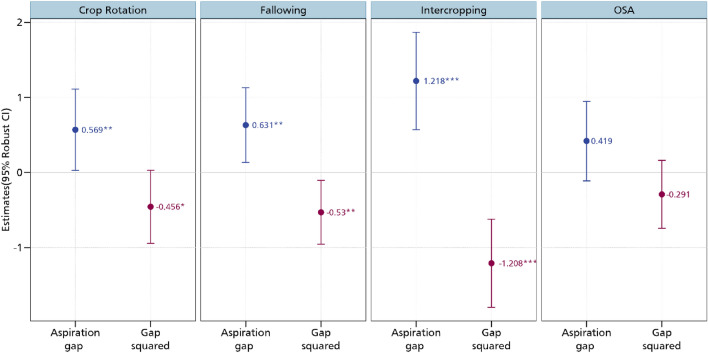
Estimates of aspiration failure and CSA practices. OSA is organic soil amendments. Additional controls include age and educational level, whether household head is male, cooperative membership, extension access, credit access, household size, off-farm employment, and wealth index. *** *p* < 0.01, ** *p* < 0.05, * *p* < 0.1. Full results are presented in Table S3 in the supplementary material.

After estimating the relationship between aspirations and the CSA practices, we now delve to the aspiration gap. As argued by^20^, it is not aspirations per se that matters in influencing future-oriented behaviours but rather the aspiration gap. Regressing this gap on the CSA practices, we establish interesting insights pertaining to aspiration failure as we imposed a quadratic term on the gap following the non-monotonic theorization^22^. Figure 4 presents the results of the relationship between the aspiration gap and the CSA practices. It also shows the relationship between the square of the aspiration gap and these practices. We observe a positive association between the aspiration gap and the adoption of crop rotation, intercropping and fallowing. Fallowing is an important agronomic practice in many smallholder farming systems that increases soil fertility especially in production systems in Cameroon where land is generally available and not a binding constraint for agricultural production. In these farming systems, agricultural production is periodically stopped for a period of time to enable nutrient restoration and better management of pests and weeds^27^.

Contrastingly, we observe a negative association between the square of aspiration gap and CSA practices. These results suggest that while aspirations are needed to stir the adoption of crop rotation, intercropping, and fallowing, overly high aspirations may result to non-adoption. These insights are in line with the theoretical prediction that aspirations that are close to the current state are the best incentives for investments^22^. Extremely high aspirations with regards to the current state may rather lead to frustration and resignation. The U-shaped tests further confirm the inverse U-shaped relationship between aspirations and adoption of CSA practices. As shown in Table S4 in the supplementary material, the Sasabuchi p-values for crop rotation, intercropping and fallowing are low though varying. This implies that the first derivative of the quadratic fit is not the same sign at both the maximum and minimum points. We also estimate turning points ranging between 0.50 and 0.70 with the Fieller confidence intervals enveloping the turning point of the U-shaped function. Our results here are largely in line with the growing literature on the inverse U-shaped relationship between aspirations and investments^21,23^. These studies have confirmed these relationship for different investment outcomes such as savings, financial decisions, educational outcomes, technology adoption, livestock, and real estate investments^17,18,31–33,35,47^.

#### Non-parametric estimation results

Although we have shown the existence of aspiration failure for the case of CSA practices employing both parametric regressions and U-shaped tests, one may argue that we imposed a functional form on how the aspiration gap enters the regression framework. To reduce these concerns, we further estimate non-parametric regressions where we do not impose any functional form. We however let the data represent the true relationship. We use loess smoothing which is a nonparametric method that uses a local weighted regression to fit a smooth curve through points in a scatter plot.

**Figure 5:**
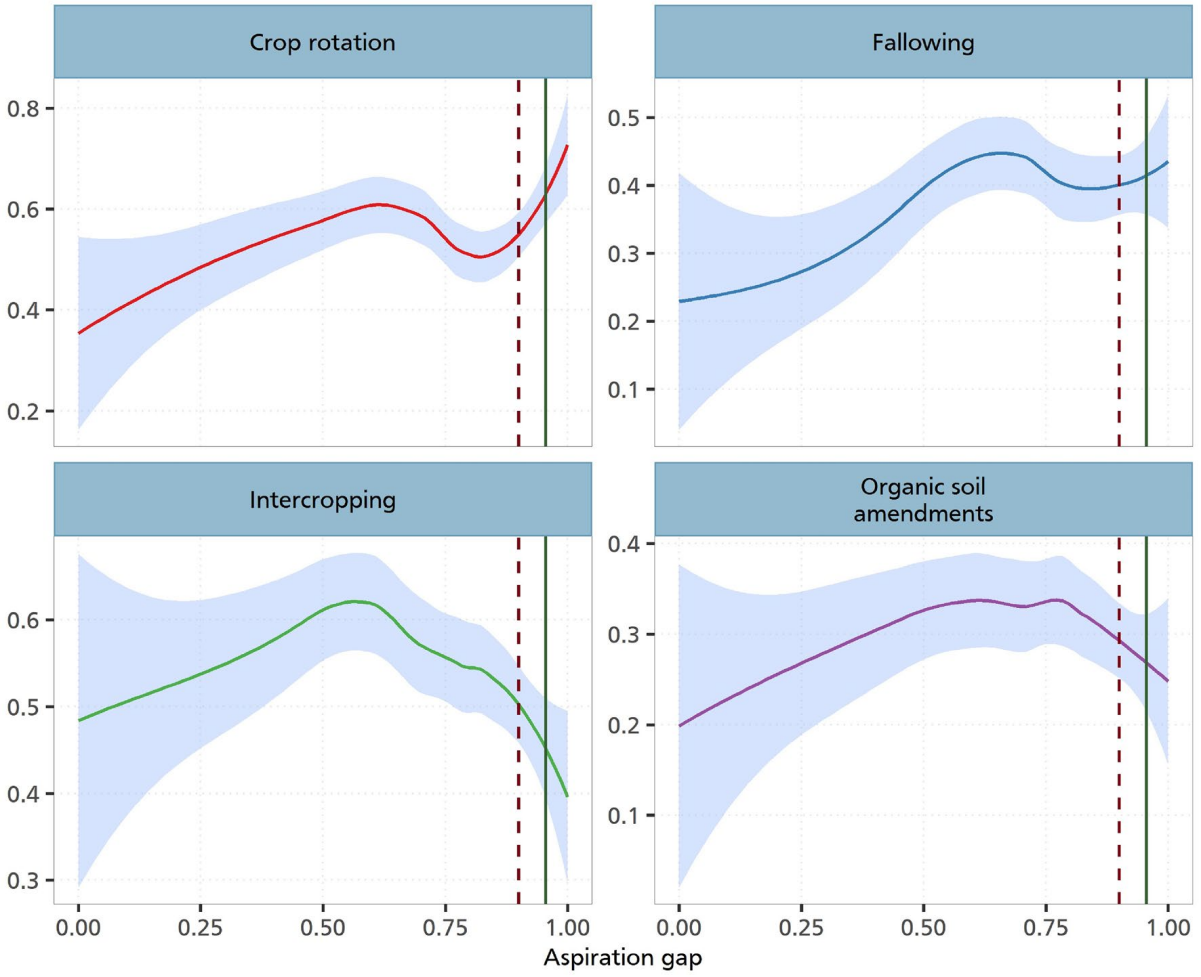
Nonparametric estimation of aspiration gaps and investments. The figure illustrates the relationship between the CSA practices and the aspiration gap. The solid line on each plot depicts a Loess smoothed relationship between aspiration gap and CSA practices. Loess smoothing is a nonparametric method that uses local weighted regression to fit a smooth curve through points in a scatter plot. This line represents the trend of CSA adoption as aspiration gap changes. The shaded region around the Loess line represents 95% confidence intervals. The dark-red dashed line indicates the 75th percentile, and the green line represents the 90th percentile of the aspiration gap. This figure is derived from a locally weighted regression analysis with a span (alpha) of 0.8 and a polynomial degree of 0.

Figure 5 shows these non-parametric estimations. As can be seen in all the various panels, we observe relationships which are in line with the previous estimations. We show that the adoption of CSA practices responds to changes in the aspiration gap. This increase is non-linear as it reaches a threshold point where it begins to decline. Given that we observe a positive association with intercropping, fallowing and the use of organic soil amendments, our analysis corroborates and provide empirical support to studies that have established a positive association between behavioural factors and the adoption of sustainable and climate-resilient farm practices^13,30^. For the case of crop rotation, we also observe a non-linear relationship, although this seems to follow an upward trajectory.

#### Cross country heterogeneity

**Figure 6:**
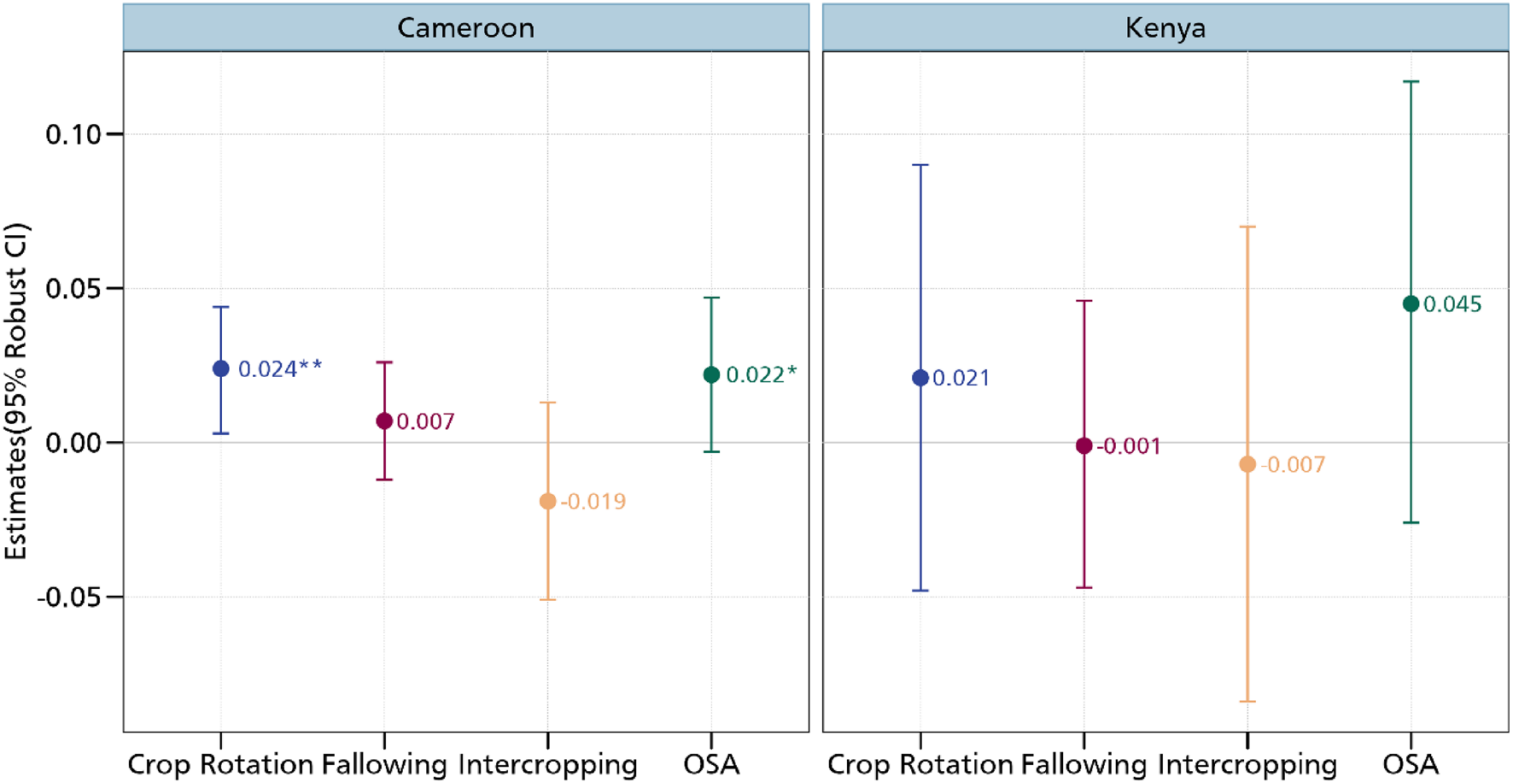
Estimates of aspirations and CSA practices in Cameroon and Kenya. OSA is organic soil amendments. Additional controls include age and educational level, whether household head is male, cooperative membership, extension access, credit access, household size, off-farm employment, and wealth index. *** *p* < 0.01, ** *p* < 0.05, * *p* < 0.1. Full results are presented in Table S5 in the supplementary material.

Now that we have shown that aspirations and the aspiration gap matter in driving investments in CSA, we perform some cross-country heterogeneity analysis where we look at the differences in the two countries. This is important given that CSA practices have been highlighted to be context specific^2^. Figure 6 shows the relationship between aspirations and CSA practices in Cameroon. Akin to the main estimations, we find that crop rotation and the use of organic soil amendments are positively associated with aspirations. No statistical relationship is again observed for intercropping and fallowing. We also present the results of the relationship between aspirations and investments in CSA practices in Kenya in  Fig. 6 where we establish no statistically significant relationship between aspirations and all the CSA practices. Figure 6 also shows these results with some consistency in signs for crop rotation and the use of organic soil amendments.

**Figure 7:**
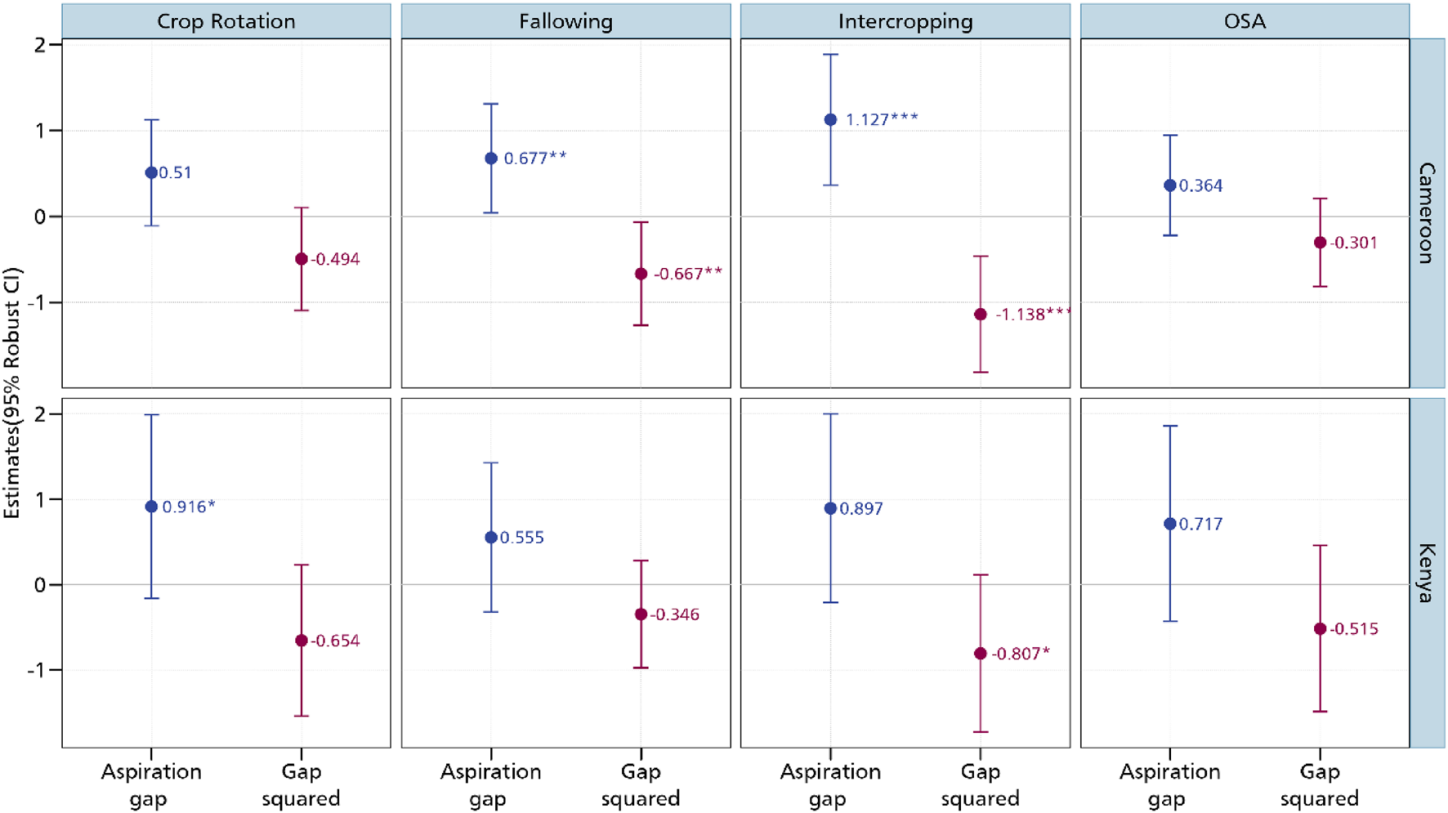
Estimates of aspiration failure and CSA practices in Cameroon and Kenya. OSA is organic soil amendments. Additional controls include age and educational level, whether household head is male, cooperative membership, extension access, credit access, household size, off-farm employment, and wealth index. *** *p* < 0.01, ** *p* < 0.05, * *p* < 0.1. Full results are presented in Table S6 in the supplementary material.

We also look at the relationship between the aspiration gap and the adoption of CSA practices in Cameroon (Fig. 7). Here, we again find a confirmation of aspiration failure with regards to investments in CSA practices. However, the results are only confirmed for intercropping and fallowing. This enables us to conclude that investments in CSA practices respond to aspiration changes among smallholder farmers in Cameroon. Figure 7 also shows the relationship between the aspiration gap and the adoption of CSA practices in Kenya. Here, we maintain the positive association between the aspiration gap and crop rotation. We obtain a negative coefficient for the relationship between the square of the aspiration gap and crop rotation, although this coefficient is not statistically significant. For intercropping, we obtain the positive and negative association with the aspiration gap and square of the aspiration gap, respectively although just the gap is statistically significant. The coefficient of the square of the aspiration is significant at the 10% level of probability, enabling us to partially conclude about the existence of an aspiration failure among smallholder farmers in Kenya.

**Figure 8:**
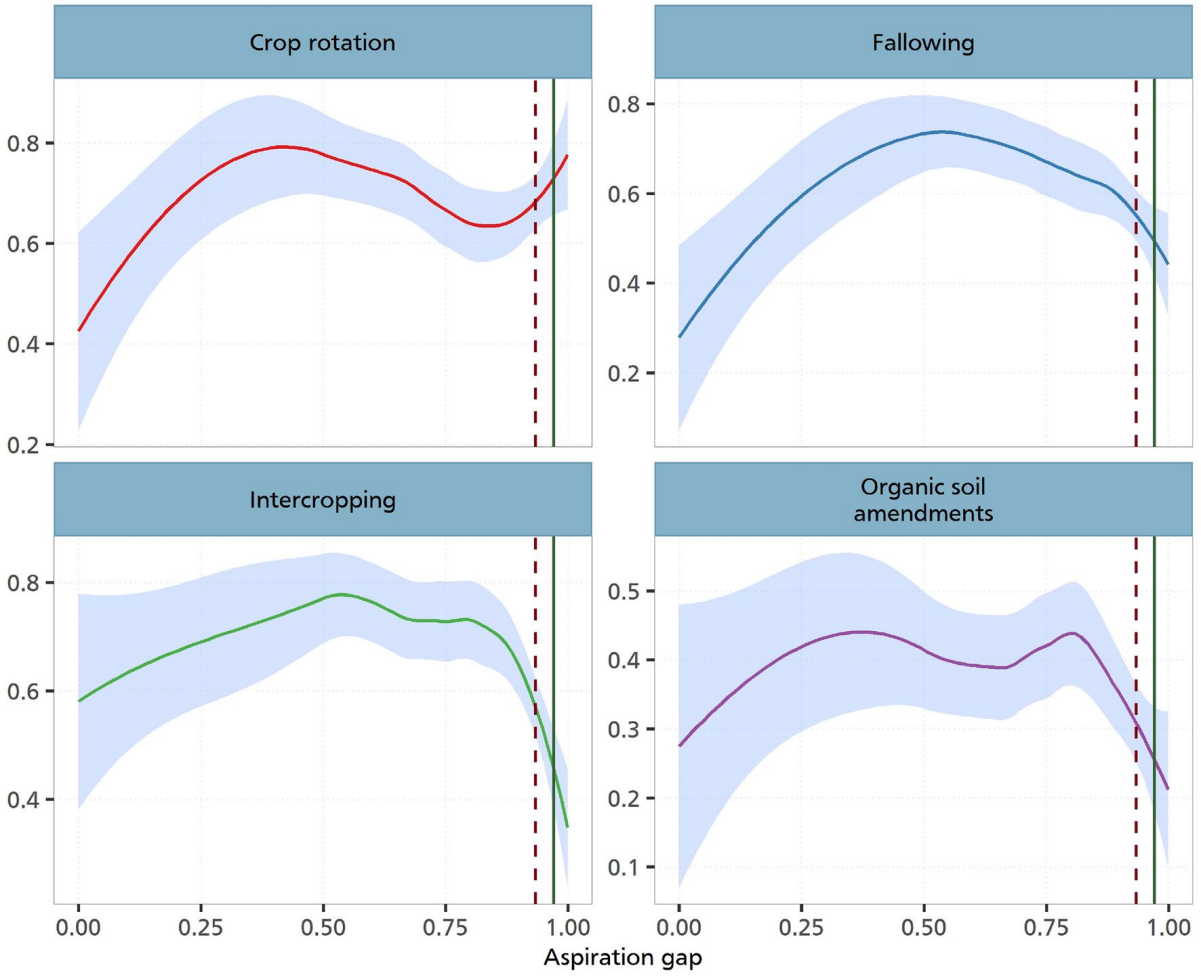
Nonparametric estimation of aspiration gaps and investments in Cameroon. The graph illustrates the relationship between the CSA practices and the aspiration gap in Cameroon. The solid line on each plot depicts a loess smoothed relationship between aspiration gap and CSA practices. Loess smoothing is a nonparametric method that uses local weighted regression to fit a smooth curve through points in a scatter plot. This line represents the trend of CSA adoption as aspiration gap changes. The shaded region around the loess line represents 95% confidence intervals. The dark-red dashed line indicates the 75th percentile, and the green line represents the 90th percentile of the aspiration gap. This figure is derived from a locally weighted regression analysis with a span (alpha) of 0.8 and a polynomial degree of 0.

**Figure 9:**
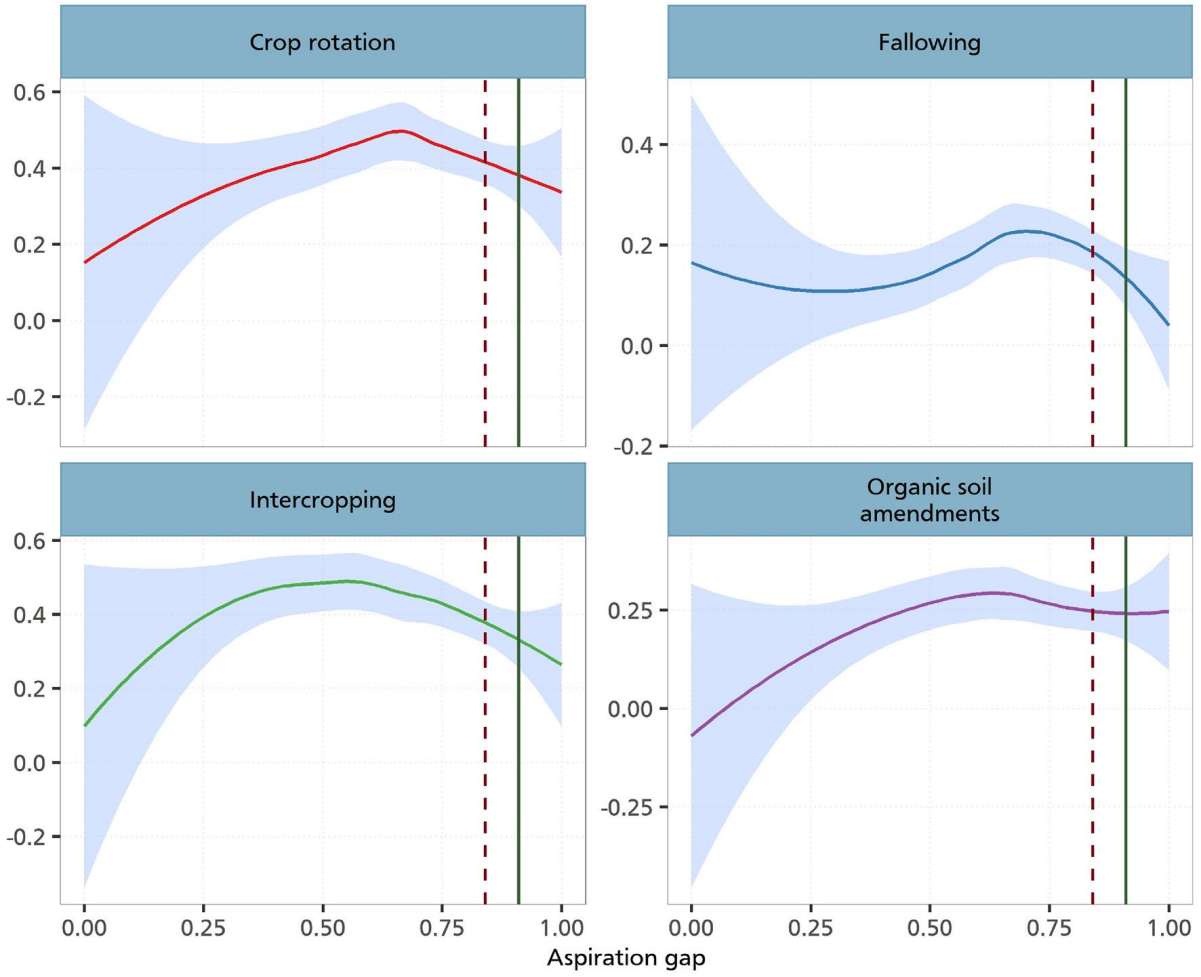
Nonparametric estimation of aspiration gaps and investments in Kenya. The graph illustrates the relationship between the CSA practices and the aspiration gap in Kenya. The solid line on each plot depicts a loess smoothed relationship between aspiration gap and CSA practices. Loess smoothing is a nonparametric method that uses local weighted regression to fit a smooth curve through points in a scatter plot. This line represents the trend of CSA adoption as aspiration gap changes. The shaded region around the loess line represents 95% confidence intervals. The dark-red dashed line indicates the 75th percentile, and the green line represents the 90th percentile of the aspiration gap. This figure is derived from a locally weighted regression analysis with a span (alpha) of 0.8 and a polynomial degree of 0.

Our non-parametric estimation for Cameroon confirms the parametric insights. The visuals here are even more reflective of this relationship. Figure 8 clearly show this increasing and subsequently decreasing relationship for fallowing, intercropping and organic soil amendments. For Kenya, we also obtain similar insights like in the case of the parametric estimations. As shown in Fig. 9, we only observe a weak non-parametric relationship between aspirations and the adoption of CSA practices.

#### Complementarities in adoption

Previous studies on adoption of sustainable and climate-resilient farm practices have shown that farmers do not adopt these practices individually^9,48^. They are usually adopted as a bundle under which they obtain the intended benefits more fully^6,49^. In this regard, we consider that the adoption of these practices may indeed be interrelated and exhibit significant interdependencies. The adoption of crop rotation may potentially exhibit complementary relationships with intercropping, fallowing, and the use of organic soil amendments as they offer similar opportunities in terms of increasing agricultural productivity and ensuring environmental sustainability. We use the multivariate probit model to capture these interdependencies as well as estimate the relationship between aspirations, aspiration gap, aspiration gap squared and the adoption of CSA practices. This model is well suited for this purpose as it estimates various error term correlations between the practices which is suggestive of complementarities or trade-offs in the use of these practices^45^.

**Figure 10:**
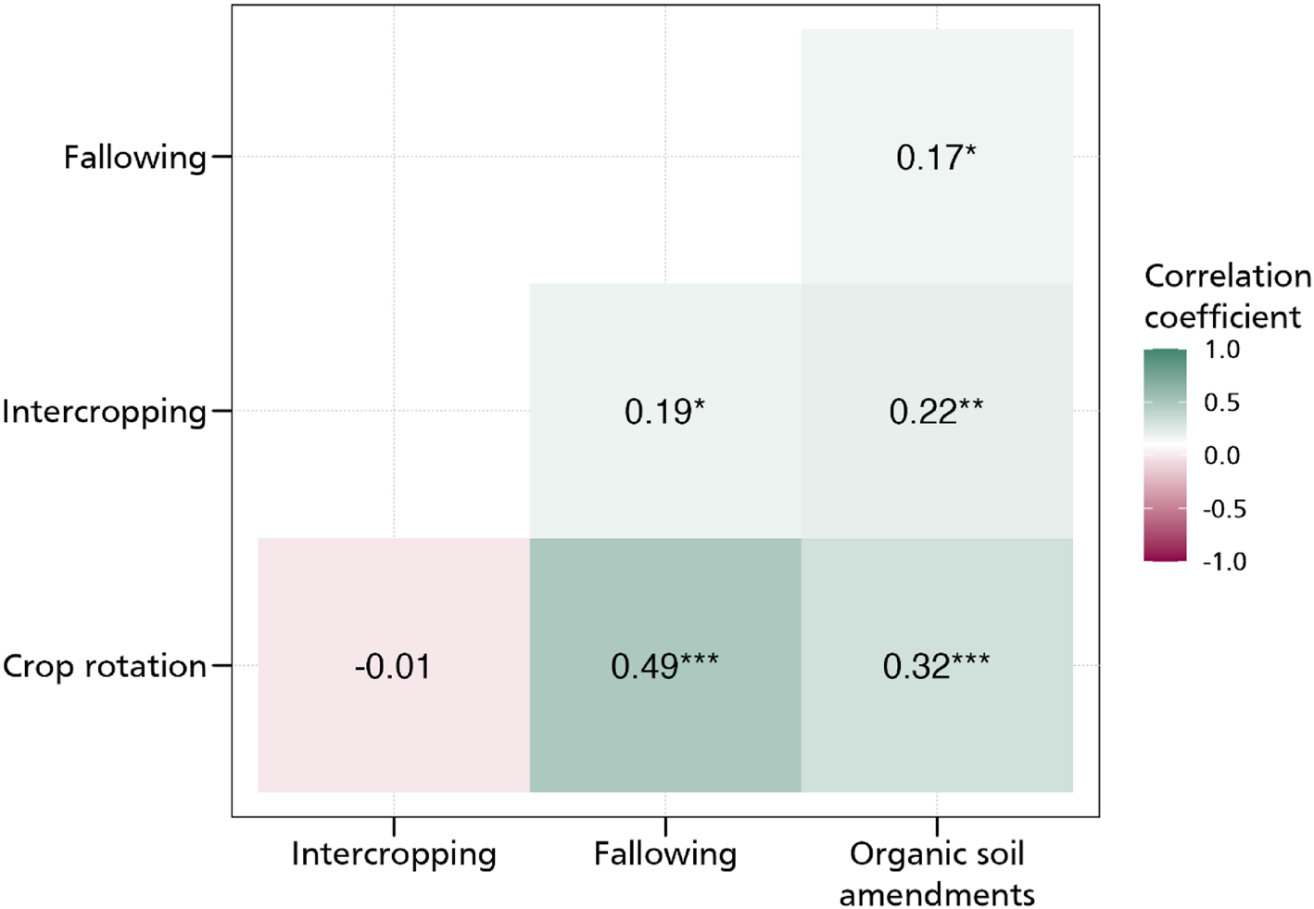
MVP of the relationship between aspirations and CSA practices. The models are estimated with additional controls such as age and educational level, whether household head is male, cooperative membership, extension access, credit access, household size, off-farm employment, and wealth index. *** *p* < 0.01, ** *p* < 0.05, * *p* < 0.1.

**Figure 11:**
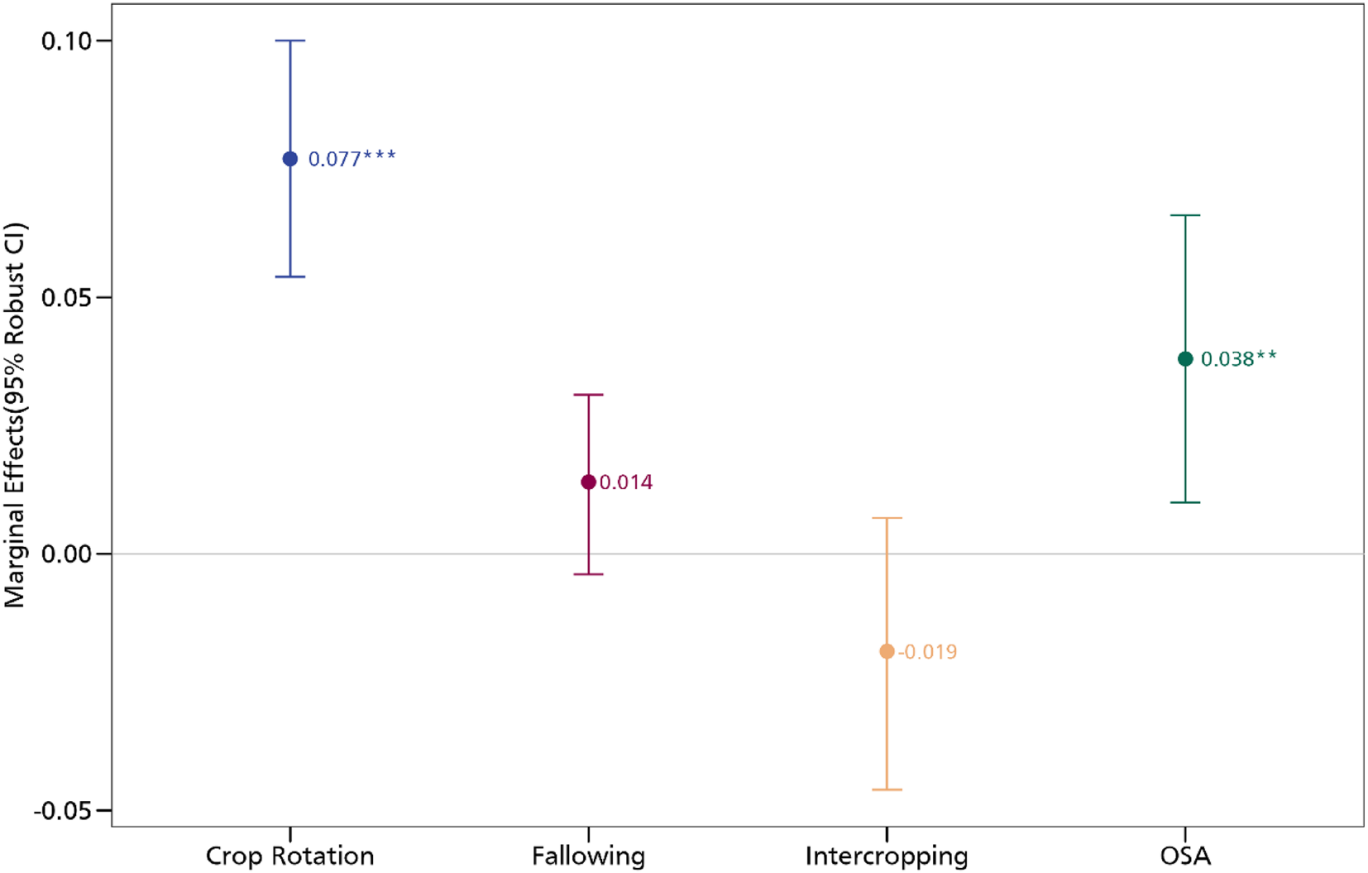
MVP estimate of the relationship between aspirations and CSA. The models are estimated with additional controls such as age and educational level, whether household head is male, cooperative membership, extension access, credit access, household size, off-farm employment, and wealth index. *** *p* < 0.01, ** *p* < 0.05, * *p* < 0.1.

**Figure 12:**
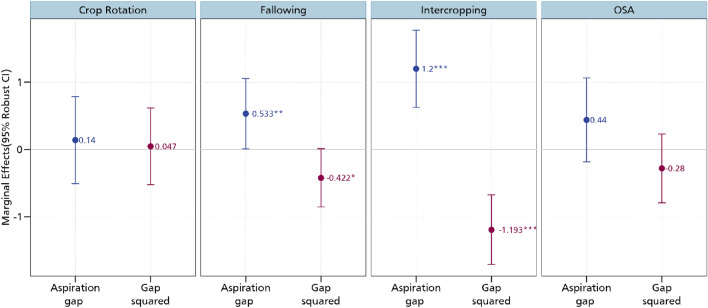
MVP estimate of the relationship between aspiration gap and CSA. The models are estimated with additional controls such as age and educational level, whether household head is male, cooperative membership, extension access, credit access, household size, off-farm employment, and wealth index. *** *p* < 0.01, ** *p* < 0.05, * *p* < 0.1.

Figure 10 shows the correlation coefficients of the relationship between the various CSA practices. While a positive correlation coefficient is indicative of a complementary relationship, a negative correlation coefficient suggests trade-offs. We obtain a positive correlation between crop rotation and fallowing, crop rotation and organic soil amendments, intercropping and fallowing, intercropping and organic soil amendments, as well as fallowing and organic soil amendments. All these positive relationships confirm that these practices are usually adopted as a bundle which may indeed earn them greater productivity, profitability, and environmental benefits. The MVP model also estimates the relationship between our aspiration measures and the CSA practices. As shown in Figs. 11 and 12 but more fully in the supplementary materials, we obtain results that support our earlier estimates (we report the marginal effects). In the supplementary materials, we present the coefficient estimates in Tables S7 and S8. Most of the magnitudes, signs, and directions of the coefficients are unchanged, enabling us to conclude that our linear estimations are in order.

For the robustness checks where we use both the Poisson regression and the ordered probit regression model, we find results that are similar and in line with the both the LPM and the MVP model. The results are presented in Tables S9 and S10 in the supplementary material for both aspirations and the aspiration gap respectively. All these further strengthen the main findings and insights from the analysis that aspirations that are ahead but not too far ahead of the current state serve as the best incentives for stirring the adoption of CSA practices.

## Conclusion and policy implications

In this study, we examine the relationship between aspirations, aspiration gaps and in the adoption of CSA practices such as crop rotation, intercropping, fallowing, and the use of organic soil amendments. We estimate both linear and nonlinear regression models in which we find a consistent and robust relationship between aspirations and the adoption of CSA practices that are climate resilient and provide multiple benefits to smallholder farmers and their environments. We also establish a similar relationship for the aspiration gap where we show a non-monotonic relationship. This non-monotonic relationship suggests the presence of aspiration failure for smallholder farmers in Cameroon and Kenya. Given that these practices may exhibit some interdependencies, we estimate a multivariate probit model that accommodates these interdependencies and show correlations between the various practices. In line with the empirical literature, we show the existence of significant complementarities in the use of these practices.

Our results provide empirical support to the vital role of behavioural factors in driving the adoption and of CSA practices. In this case, we show that aspirations which have been shown to be associated with various future-oriented economic and political behaviours are important in the adoption of CSA practices with ensuing implications on agricultural productivity and environmental sustainability. Given the numerous calls and policy talks on ways of increasing agricultural productivity and ensuring environmental sustainability, we find one key entry point for policy to leverage the adoption of CSA practices that have the potential to build adaptation to climate change through the reduced use of unsustainable farm practices. Given this, it would be important for development policy to seek ways of increasing aspirations. Some ways that have been identified include exposure to role models and media, training and edutainment programs, various social institutions where individuals can interact and learn from their peers, as well as social protection and social relief support to build the material base of households. Nudging and nurturing individual aspirations would go a long way to improve aspirations with implications for environmental sustainability.

That said, we guide against a narrow focus on aspirations as extremely high aspirations can lead to adverse economic behaviours in the light of the inverse U-shaped relationship between aspirations and investments. It is thus important to not over increase aspirations, but how to do this is an important question that we do not address in this study but is worth examining. Future studies may want to investigate this important question. One thought here would be to always consider the initial level and current state of individuals in any aspiration treatment. For households whose current states are low, the provision of big push support such as cash transfers and other social supports has a big role to play. Relatedly, it would be worthwhile to not consider aspirations as an end in themselves but rather to view them as a complementary tool to lift households out of poverty.

We end by mentioning some limitations of our study which could be taken up in future research endeavours. First, we have employed different empirical strategies and frameworks to establish a robust relationship between aspirations and the adoption of CSA practices, but we refer to all these estimations as associations with little or no implications for causality. That notwithstanding, our analysis is very informative and speaks to both theoretical and empirical literatures on the behavioural drivers of CSA practices. Future studies may want to rely on panel surveys that can control for confounders. The use of experimental methods may not be a perfect option here especially with regards to the inverse U-shaped relationship between aspirations and investments. If this relationship is true as it is increasingly highlighted, it may have some ethical concerns as it may rather reduce various outcomes of interest. Thus, reliance of cross-sectional (panel) datasets may be the way to go. Second, we have considerable external validity which may call for generalizations. However, we guide against an absolute generalization of our analysis given that context matters. That said, our results may be applicable to smallholder rural systems that face similar institutional, production and market related constraints like in Cameroon and Kenya. This would imply generalization to small farm sectors in many developing nations. However, future studies may want to build on these studies and further improve the external validity. 

### Supplementary Information

Supplementary Information.

## Data Availability

All analysis were performed on both STATA version 18 and R 4.3.1. Data and accompanying codes are freely available and can be obtained here (https://github.com/bsrthyle/behavioural-factors-and-adoption-of-CSA).
