# Supplementary material for: Behavioural factors matter for the adoption of climate-smart agriculture
Source: Sci Rep. 2024 Jan 8;14:798. doi: 10.1038/s41598-023-50264-4 (PMC10774420; doi:10.1038/s41598-023-50264-4)
Supplement: Supplementary file 1 — Supplementary Information. [file 41598_2023_50264_MOESM1_ESM.pdf]

Behavioural Factors Matter for the Adoption of Climate-Smart Agriculture

Supplementary Tables

Martin Paul Jr, Tabe-Ojong, Marvin E., Kedinga & Bisrat Haile Gebrekidan

Table of Contents

1 Descriptive statistics . . . 4

2 Main regression tables . . . 6

3 Cross country heterogeneity . . . 11

4 MVP of the relationship between aspirations, aspiration failures and CSA practices . . . 16

5 Robustness Checks . . . 21

List of Tables

S1 Descriptive statistics by country 4
S2 Full OLS estimates of the relationship between aspirations and CSA practices 6
S3 Full OLS estimates of the relationship between aspirations failure and adoption of CSA practices 8
S4 U-shaped tests of aspiration failure 10
S5 Full OLS estimates of the relationship between aspirations and CSA practices by country . 12
S6 Full OLS estimates of the relationship between aspirations failure and CSA practices by country 14
S7 MVP of the relationship between aspirations and CSA practices 17
S8 MVP of the relationship between aspiration failure and CSA practices 19
S9 Full Poisson and Ordered probit estimates of the relationship between aspiration and CSA practices 22
S10 Full Poisson and Ordered probit estimates of the relationship between aspiration filure and CSA practices 24

1 Descriptive statistics

Table S1: Descriptive statistics by country

Characteristic	**Cameroon**, N = 582	**Kenya**, N = 530	p-value
Crop rotation (1/0)	403 (69%)	223 (42%)	<0.001
Intercropping (1/0)	380 (65%)	220 (42%)	<0.001
Fallowing (1/0)	355 (61%)	90 (17%)	<0.001
Organic soil amendments (1/0)	207 (36%)	133 (25%)	<0.001
Number of CSA adopted			<0.001
0	32 (5.5%)	139 (26%)	
1	135 (23%)	202 (38%)	
2	113 (19%)	117 (22%)	
3	224 (38%)	58 (11%)	
4	78 (13%)	14 (2.6%)	
Aspirations	13.54 (1.65)	10.95 (0.95)	<0.001
Aspiration gap (0-1)	0.76 (0.22)	0.69 (0.19)	<0.001
Gap squared (0-1)	0.63 (0.27)	0.52 (0.24)	<0.001
Income (IHS)	11.74 (1.00)	9.48 (1.25)	<0.001
Off-farm activity (1/0)	169 (29%)	144 (27%)	0.5
Household size (num)	5.0 (4.2)	5.9 (2.8)	<0.001
Credit access (1/0)	108 (19%)	228 (43%)	<0.001
Age of head (years)	50 (15)	45 (16)	<0.001
Education level			<0.001
No formal education	24 (4.1%)	98 (18%)	
Primary Education	253 (43%)	172 (32%)	
Secondary Education	275 (47%)	234 (44%)	
University Education	30 (5.2%)	26 (4.9%)	
Cooperative membership (1/0)	109 (19%)	133 (25%)	0.010
Extension access (1/0)	110 (19%)	139 (26%)	0.003
Gender of head			0.8
Female	155 (27%)	137 (26%)	
Male	427 (73%)	393 (74%)	
Asset index	0.01 (1.71)	0.00 (1.85)	0.8

[1] n (%); Mean (SD)

[2] Pearson's Chi-squared test; Wilcoxon rank sum test

Note: The table above presents summary statistics of some of the regression variables by country. Two-sided t-tests were used for statistical testing, and the corresponding p-values are presented in the last column. The tests performed are Pearsons Chi-squared test for categorical variables and the Wilcoxon rank sum test for continuous variables.

2 Main regression tables

Table S2: Full OLS estimates of the relationship between aspirations and CSA practices

variables	Crop rotation (1/0)	Intercropping (1/0)	Fallowing (1/0)	Organic soil amendments (1/0)
Aspirations	0.024**	-0.021	0.005	0.029**
	(0.010)	(0.014)	(0.009)	(0.012)
	[0.019]	[0.133]	[0.605]	[0.022]
Off-farm activity (10)	0.036	-0.048	0.026	-0.032
	(0.037)	(0.039)	(0.032)	(0.032)
	[0.338]	[0.225]	[0.420]	[0.317]
Household size (num)	0.006	0.004	0.001	0.001
	(0.004)	(0.005)	(0.004)	(0.004)
	[0.155]	[0.346]	[0.699]	[0.773]
Credit access (10)	0.119***	0.060*	0.031	0.009
	(0.036)	(0.031)	(0.031)	(0.036)
	[0.001]	[0.061]	[0.314]	[0.798]
Age of head (years)	0.000	0.000	-0.001	-0.002
	(0.001)	(0.001)	(0.001)	(0.001)
	[0.807]	[0.879]	[0.601]	[0.189]
Educational level (years)	-0.002	0.001	-0.006*	-0.006
	(0.004)	(0.005)	(0.003)	(0.004)
	[0.654]	[0.904]	[0.071]	[0.153]
Cooperative membership (10)	0.039	0.028	0.041	0.019
	(0.037)	(0.036)	(0.033)	(0.035)
	[0.292]	[0.436]	[0.217]	[0.584]
Extension access (10)	0.085**	0.071*	-0.005	0.046
	(0.041)	(0.040)	(0.033)	(0.031)
	[0.043]	[0.077]	[0.877]	[0.139]
Head is male (10)	0.009	0.025	-0.014	0.001
	(0.031)	(0.039)	(0.028)	(0.030)
	[0.780]	[0.523]	[0.616]	[0.974]
Asset index	0.035***	-0.012	0.038***	0.034***
	(0.012)	(0.017)	(0.011)	(0.013)
	[0.006]	[0.489]	[0.001]	[0.008]
Observations	1,112	1,112	1,112	1,112
R-squared	0.342	0.239	0.388	0.226
F test	5.748	1.653	2.606	3.905

Note: The table presents the results of OLS regressions between aspirations and CSA practices , with robust standard errors, where the standard errors are clustered. The statistical tests conducted are two-sided t-tests. P-values are denoted in square brackets. The presence of an asterisk (*) above a coefficient indicates that the coefficient is statistically different from zero at a predetermined level of significance (*** p<0.01, ** p<0.05, * p<0.1)

Table S3: Full OLS estimates of the relationship between aspirations failure and adoption of CSA practices

variables	Crop rotation (1/0)	Intercropping (1/0)	Fallowing (1/0)	Organic soil amendments (1/0)
Aspiration gap (0-1)	0.569**	1.218***	0.631**	0.419
	(0.271)	(0.325)	(0.250)	(0.266)
	[0.040]	[0.000]	[0.014]	[0.120]
Gap squared (0-1)	-0.456*	-1.208***	-0.530**	-0.291
	(0.244)	(0.294)	(0.212)	(0.227)
	[0.065]	[0.000]	[0.015]	[0.204]
Off-farm activity (10)	0.036	-0.064*	0.024	-0.029
	(0.038)	(0.038)	(0.033)	(0.032)
	[0.352]	[0.097]	[0.480]	[0.363]
Household size (num)	0.006	0.004	0.002	0.002
	(0.004)	(0.004)	(0.004)	(0.004)
	[0.130]	[0.308]	[0.659]	[0.715]
Credit access (10)	0.117***	0.057*	0.030	0.007
	(0.036)	(0.032)	(0.031)	(0.036)
	[0.002]	[0.083]	[0.338]	[0.843]
Age of head (years)	0.000	0.000	-0.001	-0.002
	(0.001)	(0.001)	(0.001)	(0.001)
	[0.947]	[0.990]	[0.550]	[0.153]
Educational level (years)	-0.001	-0.001	-0.006*	-0.005
	(0.004)	(0.005)	(0.003)	(0.004)
	[0.789]	[0.840]	[0.071]	[0.223]
Cooperative membership (10)	0.035	0.009	0.036	0.018
	(0.037)	(0.035)	(0.031)	(0.035)
	[0.348]	[0.804]	[0.254]	[0.596]
Extension access (10)	0.086**	0.078*	-0.004	0.045
	(0.041)	(0.040)	(0.033)	(0.031)
	[0.041]	[0.053]	[0.893]	[0.142]
Head is male (10)	0.008	0.012	-0.018	0.002
	(0.031)	(0.039)	(0.028)	(0.030)
	[0.802]	[0.754]	[0.532]	[0.943]
Asset index	0.039***	-0.016	0.039***	0.039***
	(0.012)	(0.016)	(0.011)	(0.012)
	[0.002]	[0.334]	[0.001]	[0.002]
Observations	1,112	1,112	1,112	1,112
R-squared	0.341	0.262	0.392	0.222
F test	6.342	3.600	2.849	3.614

Note: The table presents the results of OLS regressions between aspirations failure and CSA practices. Robust standard errors are in brackates. The statistical tests conducted are two-sided t-tests. P-values, denoted in square brackets. The presence of an asterisk (*) above a coefficient indicates that the coefficient is statistically different from zero at a predetermined level of significance (*** p<0.01, ** p<0.05, * p<0.1).

Table S4: U-shaped tests of aspiration failure

	**Crop rotation**	**Intercropping**	**Fallowing**	**OSA**
**[A] Pooled**				
Turning point	0.623	0.504	0.595	0.718
Sasabuchi p-value	0.071	<0.001	0.016	0.215
Slope at minimum	0.568	1.218	0.631	0.418
Slope at maximum	-0.344	-1.196	-0.428	-0.163
Fieller 95% confidence interval	[-Inf; +Inf]	[0.419; 0.569]	[0.427; 0.86]	[-Inf; +Inf]
**[B] Cameroon**				
Turning point	0.515	0.494	0.507	0.604
Sasabuchi p-value	0.067	0.002	0.019	0.169
Slope at minimum	0.509	1.126	0.676	0.363
Slope at maximum	-0.478	-1.149	-0.656	-0.238
Fieller 95% confidence interval	[-Inf; +Inf]	[0.356; 0.577]	[0.21; 0.834]	[-Inf; +Inf]
**[C] Kenya**				
Turning point	0.7	0.555	0.803	0.696
Sasabuchi p-value	0.142	0.054	0.267	0.229
Slope at minimum	0.916	0.897	0.555	0.717
Slope at maximum	-0.392	-0.716	-0.136	-0.312
Fieller 95% confidence interval	[-Inf; +Inf]	[-Inf; +Inf]	[-Inf; +Inf]	[-Inf; +Inf]

3 Cross country heterogeneity

Table S5: Full OLS estimates of the relationship between aspirations and CSA practices by country

	Cameroon				Kenya			
variables	Crop rotation (1/0)	Intercropping (1/0)	Fallowing (1/0)	Organic soil amendments (1/0)	Crop rotation (1/0)	Intercropping (1/0)	Fallowing (1/0)	Organic soil amendments (1/0)
Aspirations	0.024**	-0.019	0.007	0.022*	0.021	-0.007	-0.001	0.045
	(0.010)	(0.016)	(0.009)	(0.012)	(0.034)	(0.038)	(0.023)	(0.035)
	[0.024]	[0.241]	[0.457]	[0.081]	[0.535]	[0.856]	[0.981]	[0.207]
Off-farm activity (10)	0.025	-0.068	-0.027	-0.044	0.059	-0.024	0.094**	0.002
	(0.057)	(0.055)	(0.045)	(0.044)	(0.048)	(0.051)	(0.045)	(0.047)
	[0.661]	[0.226]	[0.549]	[0.324]	[0.231]	[0.645]	[0.045]	[0.969]
Household size (num)	0.003	-0.001	0.002	0.001	0.013	0.011	0.001	0.002
	(0.004)	(0.004)	(0.004)	(0.005)	(0.008)	(0.009)	(0.007)	(0.008)
	[0.508]	[0.878]	[0.678]	[0.852]	[0.138]	[0.245]	[0.913]	[0.838]
Credit access (10)	0.122**	0.032	0.121**	0.016	0.120**	0.078*	-0.019	0.014
	(0.057)	(0.040)	(0.053)	(0.064)	(0.049)	(0.043)	(0.032)	(0.042)
	[0.038]	[0.428]	[0.030]	[0.798]	[0.020]	[0.081]	[0.554]	[0.730]
Age of head (years)	-0.000	-0.002	-0.002	-0.003	0.001	0.002	0.000	0.000
	(0.001)	(0.002)	(0.001)	(0.002)	(0.002)	(0.002)	(0.002)	(0.002)
	[0.797]	[0.147]	[0.159]	[0.120]	[0.580]	[0.339]	[0.779]	[0.943]
Educational level (years)	0.000	0.004	0.003	-0.007	-0.003	-0.006	-0.010**	-0.003
	(0.005)	(0.007)	(0.005)	(0.006)	(0.007)	(0.006)	(0.005)	(0.005)
	[0.986]	[0.602]	[0.591]	[0.236]	[0.649]	[0.316]	[0.040]	[0.472]
Cooperative membership (10)	0.009	0.042	0.068	0.090*	0.085	-0.015	0.027	-0.034
	(0.049)	(0.040)	(0.050)	(0.050)	(0.057)	(0.058)	(0.042)	(0.051)
	[0.852]	[0.299]	[0.185]	[0.082]	[0.149]	[0.803]	[0.518]	[0.507]
Extension access (10)	-0.005	0.041	-0.068	-0.057	0.161***	0.114**	0.044	0.118***
	(0.060)	(0.059)	(0.049)	(0.042)	(0.059)	(0.048)	(0.046)	(0.036)
	[0.931]	[0.487]	[0.180]	[0.184]	[0.010]	[0.024]	[0.340]	[0.002]
Head is male (10)	-0.001	-0.031	-0.039	-0.050	0.031	0.030	0.032	0.087*
	(0.042)	(0.055)	(0.034)	(0.037)	(0.045)	(0.052)	(0.043)	(0.045)
	[0.980]	[0.571]	[0.260]	[0.186]	[0.491]	[0.573]	[0.465]	[0.059]
Asset index	0.043**	-0.083***	0.045**	0.060***	0.022	0.044**	0.033**	0.005
	(0.018)	(0.022)	(0.018)	(0.021)	(0.017)	(0.018)	(0.015)	(0.016)
	[0.026]	[0.001]	[0.018]	[0.006]	[0.194]	[0.020]	[0.035]	[0.776]
Observations	582	582	582	582	530	530	530	530
R-squared	0.311	0.189	0.315	0.323	0.282	0.307	0.125	0.114
Additional controls	Yes	Yes	Yes	Yes	Yes	Yes	Yes	Yes
Village FE	No	No	No	No	Yes	Yes	Yes	Yes
F test	2.215	4.160	2.803	2.433	7.010	4.657	2.372	3.224

Note: The table presents the results of OLS regressions between aspirations failure and CSA practices by country.Robust standard errors are in brackates. The statistical tests conducted are two-sided t-tests. P-values is denoted in square brackets. The presence of an asterisk (*) above a coefficient indicates that the coefficient is statistically different from zero at a predetermined level of significance (*** p<0.01, ** p<0.05, * p<0.1). All regressions include a comprehensive set of village fixed effects to control for potential unobserved heterogeneity.

Table S6: Full OLS estimates of the relationship between aspirations failure and CSA practices by country

	Cameroon				Kenya			
variables	Crop rotation (1/0)	Intercropping (1/0)	Fallowing (1/0)	Organic soil amendments (1/0)	Crop rotation (1/0)	Intercropping (1/0)	Fallowing (1/0)	Organic soil amendments (1/0)
Aspiration gap (0-1)	0.510	1.127***	0.677**	0.364	0.916*	0.897	0.555	0.717
	(0.304)	(0.375)	(0.311)	(0.288)	(0.529)	(0.544)	(0.431)	(0.565)
	[0.102]	[0.005]	[0.037]	[0.214]	[0.092]	[0.108]	[0.206]	[0.213]
Gap squared (0-1)	-0.494	-1.138***	-0.667**	-0.301	-0.654	-0.807*	-0.346	-0.515
	(0.295)	(0.333)	(0.296)	(0.252)	(0.437)	(0.454)	(0.310)	(0.480)
	[0.103]	[0.002]	[0.031]	[0.240]	[0.143]	[0.084]	[0.272]	[0.291]
Off-farm activity (10)	0.028	-0.071	-0.026	-0.042	0.067	-0.035	0.106**	0.006
	(0.057)	(0.054)	(0.047)	(0.044)	(0.048)	(0.049)	(0.049)	(0.048)
	[0.621]	[0.195]	[0.579]	[0.347]	[0.178]	[0.486]	[0.037]	[0.903]
Household size (num)	0.003	0.000	0.002	0.001	0.013	0.010	0.001	0.002
	(0.004)	(0.004)	(0.004)	(0.005)	(0.008)	(0.009)	(0.007)	(0.008)
	[0.421]	[0.966]	[0.587]	[0.799]	[0.124]	[0.264]	[0.842]	[0.821]
Credit access (10)	0.119**	0.030	0.118**	0.013	0.118**	0.077*	-0.019	0.012
	(0.056)	(0.039)	(0.053)	(0.065)	(0.050)	(0.044)	(0.031)	(0.043)
	[0.041]	[0.460]	[0.035]	[0.838]	[0.023]	[0.093]	[0.542]	[0.789]
Age of head (years)	-0.001	-0.002	-0.002	-0.003	0.001	0.002	0.001	0.000
	(0.001)	(0.001)	(0.001)	(0.002)	(0.002)	(0.002)	(0.002)	(0.002)
	[0.702]	[0.128]	[0.123]	[0.106]	[0.527]	[0.373]	[0.652]	[0.973]
Educational level (years)	-0.001	0.002	0.001	-0.008	-0.001	-0.006	-0.010**	-0.000
	(0.005)	(0.007)	(0.005)	(0.006)	(0.006)	(0.006)	(0.005)	(0.005)
	[0.913]	[0.830]	[0.766]	[0.211]	[0.860]	[0.325]	[0.046]	[0.939]
Cooperative membership (10)	-0.001	0.013	0.052	0.086*	0.085	-0.016	0.029	-0.036
	(0.049)	(0.040)	(0.046)	(0.050)	(0.059)	(0.057)	(0.041)	(0.052)
	[0.989]	[0.750]	[0.260]	[0.091]	[0.158]	[0.778]	[0.478]	[0.494]
Extension access (10)	-0.012	0.037	-0.073	-0.061	0.159**	0.119**	0.037	0.123***
	(0.060)	(0.057)	(0.050)	(0.043)	(0.059)	(0.050)	(0.043)	(0.036)
	[0.846]	[0.524]	[0.152]	[0.162]	[0.011]	[0.022]	[0.396]	[0.002]
Head is male (10)	0.004	-0.036	-0.038	-0.045	0.023	0.019	0.027	0.082*
	(0.043)	(0.055)	(0.035)	(0.036)	(0.045)	(0.052)	(0.041)	(0.045)
	[0.935]	[0.518]	[0.280]	[0.217]	[0.611]	[0.717]	[0.510]	[0.081]
Asset index	0.051***	-0.078***	0.051***	0.066***	0.024	0.039**	0.033**	0.011
	(0.018)	(0.020)	(0.018)	(0.020)	(0.016)	(0.017)	(0.014)	(0.016)
	[0.007]	[0.000]	[0.009]	[0.002]	[0.142]	[0.029]	[0.030]	[0.506]
Observations	582	582	582	582	530	530	530	530
R-squared	0.311	0.216	0.324	0.320	0.286	0.313	0.130	0.112
F test	2.570	4.618	3.005	2.023	6.858	5.829	2.632	3.818

Note: The table presents the results of OLS regressions between aspirations failure and CSA practices by country.Robust standard errors are in brackates. The statistical tests conducted are two-sided t-tests. P-values is denoted in square brackets. The presence of an asterisk (*) above a coefficient indicates that the coefficient is statistically different from zero at a predetermined level of significance (*** p<0.01, ** p<0.05, * p<0.1). All regressions include a comprehensive set of district fixed effects to control for potential unobserved heterogeneity.

4 MVP of the relationship between aspirations, aspiration failures and CSA practices

Table S7: MVP of the relationship between aspirations and CSA practices

variables	Crop rotation (1/0)	Intercropping (1/0)	Fallowing (1/0)	Organic soil amendments (1/0)
Aspirations	0.216***	-0.0594	0.0462	0.125***
	(0.0370)	(0.0421)	(0.0302)	(0.0470)
Off-farm activity (10)	-0.0803	-0.155	0.0792	-0.140
	(0.103)	(0.117)	(0.126)	(0.111)
Household size (num)	0.0135	0.0151	0.00975	0.00650
	(0.0139)	(0.0137)	(0.0145)	(0.0142)
Credit access (10)	0.224**	0.209**	0.0750	0.000383
	(0.0997)	(0.0986)	(0.115)	(0.114)
Age of head (years)	0.00227	0.000528	-0.000785	-0.00565
	(0.00301)	(0.00399)	(0.00396)	(0.00440)
Educational level (years)	-0.0129	0.00122	-0.0274*	-0.0179
	(0.0119)	(0.0143)	(0.0145)	(0.0136)
Cooperative membership (10)	0.264**	0.0867	0.227*	0.0815
	(0.105)	(0.112)	(0.126)	(0.112)
Extension access (10)	0.284**	0.228*	-0.0331	0.148
	(0.119)	(0.121)	(0.126)	(0.0988)
Head is male (10)	0.0140	0.0871	-0.0357	0.0258
	(0.0823)	(0.117)	(0.110)	(0.115)
Asset index	0.0826**	-0.0349	0.163***	0.106***
	(0.0324)	(0.0481)	(0.0429)	(0.0389)
Constant	-2.734***	0.962	-0.266	-1.510**
	(0.511)	(0.695)	(0.547)	(0.701)
Observations	1,112	1,112	1,112	1,112
Additional controls	Yes	Yes	Yes	Yes
Village FE	Yes	Yes	Yes	Yes
Year FE	Yes	Yes	Yes	Yes
_	Coefficent	Std. Err.		
atanhrho-12	-0.0102	(0.0785)		
atanhrho-13	0.491***	(0.0989)		
atanhrho-14	0.324***	(0.0855)		
atanhrho-23	0.186*	(0.0996)		
atanhrho-24	0.217**	(0.0844)		
atanhrho-34	0.173*	(0.0929)		

Robust standard errors in parentheses
*** p<0.01, ** p<0.05, * p<0.1

Note: This table displays the findings of Multivariate Probit (MVP) regressions, applied to investigate the relationship between aspiration and the adoption of Climate-Smart Agriculture (CSA) practices. Robust standard errors are reported in brackets to control for potential heteroscedasticity. Two-sided t-tests were conducted for the statistical tests, and corresponding p-values are noted within square brackets. Coefficients denoted with an asterisk () represent statistical significance at pre-established levels (** p<0.01, ** p<0.05, * p<0.1). To account for potential unobserved heterogeneity, all regressions incorporate a comprehensive set of village fixed effects.

Table S8: MVP of the relationship between aspiration failure and CSA practices

variables	Crop rotation (1/0)	Intercropping (1/0)	Fallowing (1/0)	Organic soil amendments (1/0)
Aspiration gap (0-1)	0.373	3.829***	1.783**	1.429
	(0.879)	(0.972)	(0.903)	(1.040)
Gap squared (0-1)	0.125	-3.805***	-1.413*	-0.910
	(0.776)	(0.887)	(0.747)	(0.850)
Off-farm activity (10)	-0.0336	-0.210*	0.0802	-0.126
	(0.103)	(0.118)	(0.128)	(0.110)
Household size (num)	0.00890	0.0163	0.00903	0.00687
	(0.0125)	(0.0130)	(0.0146)	(0.0138)
Credit access (10)	0.0707	0.216**	0.0350	-0.0348
	(0.105)	(0.105)	(0.114)	(0.114)
Age of head (years)	0.00438	0.000192	-0.000128	-0.00549
	(0.00308)	(0.00398)	(0.00393)	(0.00427)
Educational level (years)	0.0160	-0.00313	-0.0207	-0.00972
	(0.0114)	(0.0148)	(0.0141)	(0.0131)
Cooperative membership (10)	0.289***	0.0240	0.212*	0.0859
	(0.107)	(0.112)	(0.122)	(0.110)
Extension access (10)	0.240**	0.256**	-0.0394	0.138
	(0.118)	(0.123)	(0.125)	(0.0960)
Head is male (10)	0.0292	0.0462	-0.0454	0.0355
	(0.0878)	(0.122)	(0.111)	(0.113)
Asset index	0.110***	-0.0445	0.168***	0.125***
	(0.0305)	(0.0449)	(0.0417)	(0.0369)
Constant	-0.732**	-0.410	-0.305	-0.506
	(0.348)	(0.406)	(0.436)	(0.486)
Observations	1,112	1,112	1,112	1,112
Additional controls	Yes	Yes	Yes	Yes
Village FE	Yes	Yes	Yes	Yes
Year FE	Yes	Yes	Yes	Yes
_	Coefficent	Std. Err.		
atanhrho-12	-0.0154	(0.0822)		
atanhrho-13	0.487***	(0.0988)		
atanhrho-14	0.330***	(0.0871)		
atanhrho-23	0.173*	(0.101)		
atanhrho-24	0.209**	(0.0852)		
atanhrho-34	0.170*	(0.0924)		

Robust standard errors in parentheses
*** p<0.01, ** p<0.05, * p<0.1

Note: The table displays the findings of Multivariate Probit (MVP) regressions, applied to investigate the relationship between aspiration failure and the adoption of Climate-Smart Agriculture (CSA) practices. Robust standard errors are reported in brackets to control for potential heteroscedasticity. Two-sided t-tests were conducted for the statistical tests, and corresponding p-values are noted within square brackets. Coefficients denoted with an asterisk () represent statistical significance at pre-established levels (** p<0.01, ** p<0.05, * p<0.1). To account for potential unobserved heterogeneity, all regressions incorporate a comprehensive set of village fixed effects.

5 Robustness Checks

Table S9: Full Poisson and Ordered probit estimates of the relationship between aspiration and CSA practices

	POISSON			ORDERED PROBIT		
variable	(1)	(2)	(3)	(1)	(2)	(3)
Aspirations	0.131***	0.124***	0.021	0.210***	0.202***	0.039
	(0.019)	(0.018)	(0.014)	(0.031)	(0.030)	(0.028)
	[0.000]	[0.000]	[0.141]	[0.000]	[0.000]	[0.164]
Off-farm activity		-0.024	-0.013		-0.025	0.010
		(0.040)	(0.042)		(0.067)	(0.084)
		[0.557]	[0.756]		[0.709]	[0.906]
Household size		0.005	0.007		0.008	0.017
		(0.005)	(0.005)		(0.010)	(0.012)
		[0.381]	[0.174]		[0.418]	[0.143]
Credit access		0.024	0.128***		0.031	0.254***
		(0.045)	(0.044)		(0.075)	(0.085)
		[0.599]	[0.003]		[0.680]	[0.003]
Age of head		0.005***	-0.001		0.009***	-0.002
		(0.002)	(0.002)		(0.003)	(0.003)
		[0.003]	[0.606]		[0.002]	[0.548]
Educational level		0.002	-0.005		0.005	-0.012
		(0.006)	(0.006)		(0.011)	(0.012)
		[0.723]	[0.404]		[0.636]	[0.319]
Cooperative membership		0.035	0.074**		0.056	0.155**
		(0.042)	(0.038)		(0.074)	(0.077)
		[0.409]	[0.048]		[0.452]	[0.044]
Extension access		0.083*	0.106**		0.131	0.201**
		(0.048)	(0.051)		(0.084)	(0.102)
		[0.081]	[0.040]		[0.119]	[0.049]
Head is male		-0.006	0.017		-0.029	0.016
		(0.048)	(0.040)		(0.082)	(0.077)
		[0.895]	[0.671]		[0.720]	[0.833]
Asset index		0.047***	0.051***		0.097***	0.108***
		(0.014)	(0.017)		(0.026)	(0.036)
		[0.001]	[0.003]		[0.000]	[0.002]
Observations	1,112	1,112	1,112	1,112	1,112	1,112
Additional controls	No	Yes	Yes	No	Yes	Yes
Village FE	No	No	Yes	No	No	Yes

Note: The table provides the findings from Full Poisson and Ordered probit estimations, carried out to explore the association between aspiration and Climate-Smart Agriculture (CSA) practices. The Full Poisson model was utilized to handle count outcomes while the Ordered probit model was used for ordinal outcomes. Robust standard errors, stated within brackets, were utilized to mitigate the impact of heteroscedasticity. Statistical tests were performed using two-sided t-tests, and the corresponding p-values are displayed within square brackets. Coefficients designated with an asterisk () indicate statistical significance at preset significance thresholds (** p<0.01, ** p<0.05, * p<0.1).

Table S10: Full Poisson and Ordered probit estimates of the relationship between aspiration filure and CSA practices

	POISSON	ORDERED PROBIT
variables	(1)	(2)
Aspiration gap (0-1)	1.729***	3.251***
	(0.461)	(0.774)
	[0.000]	[0.000]
Gap squared (0-1)	-1.493***	-2.873***
	(0.364)	(0.664)
	[0.000]	[0.000]
Off-farm activity (10)	-0.017	-0.010
	(0.042)	(0.086)
	[0.677]	[0.909]
Household size (num)	0.007	0.018
	(0.005)	(0.012)
	[0.129]	[0.116]
Credit access (10)	0.123***	0.247***
	(0.044)	(0.089)
	[0.005]	[0.005]
Age of head (years)	-0.001	-0.003
	(0.002)	(0.003)
	[0.505]	[0.420]
Educational level (years)	-0.006	-0.012
	(0.006)	(0.013)
	[0.351]	[0.325]
Cooperative membership (10)	0.055	0.121
	(0.036)	(0.074)
	[0.123]	[0.103]
Extension access (10)	0.107**	0.214**
	(0.052)	(0.105)
	[0.041]	[0.041]
Head is male (10)	0.010	-0.001
	(0.039)	(0.077)
	[0.804]	[0.984]
Asset index	0.057***	0.116***
	(0.016)	(0.033)
	[0.000]	[0.000]
Observations	1,112	1,112
Additional controls	Yes	Yes

Village FE	Yes	Yes

Note: The table provides the findings from Full Poisson and Ordered probit estimations, carried out to explore the association between aspiration failure and Climate-Smart Agriculture (CSA) practices. The Full Poisson model was utilized to handle count outcomes while the Ordered probit model was used for ordinal outcomes. Robust standard errors, stated within brackets, were utilized to mitigate the impact of heteroscedasticity. Statistical tests were performed using two-sided t-tests, and the corresponding p-values are displayed within square brackets. Coefficients designated with an asterisk () indicate statistical significance at preset significance thresholds (** p<0.01, ** p<0.05, * p<0.1).
